# Population mobility associated with higher risk sexual behaviour in eastern African communities participating in a Universal Testing and Treatment trial

**DOI:** 10.1002/jia2.25115

**Published:** 2018-07-19

**Authors:** Carol S Camlin, Adam Akullian, Torsten B Neilands, Monica Getahun, Patrick Eyul, Irene Maeri, Sarah Ssali, Elvin Geng, Monica Gandhi, Craig R Cohen, Moses R Kamya, Thomas Odeny, Elizabeth A Bukusi, Edwin D Charlebois

**Affiliations:** ^1^ Department of Obstetrics, Gynecology & Reproductive Sciences University of California San Francisco (UCSF) San Francisco CA USA; ^2^ Department of Medicine Center for AIDS Prevention Studies UCSF San Francisco CA USA; ^3^ Institute for Disease Modeling University of Washington Seattle Washington USA; ^4^ Infectious Diseases Research Collaboration (IDRC) Makerere University (MU‐UCSF) Kampala Uganda; ^5^ Kenya Medical Research Institute (KEMRI) Nairobi Kenya; ^6^ Department of Medicine Division of HIV, Infectious Diseases and Global Medicine UCSF San Francisco CA USA

**Keywords:** HIV, universal test and treat, population dynamics, geographic mobility, sexual behaviour, sub‐Saharan Africa

## Abstract

**Introduction:**

There are significant knowledge gaps concerning complex forms of mobility emergent in sub‐Saharan Africa, their relationship to sexual behaviours, HIV transmission, and how sex modifies these associations. This study, within an ongoing test‐and‐treat trial (SEARCH, NCT01864603), sought to measure effects of diverse metrics of mobility on behaviours, with attention to gender.

**Methods:**

Cross‐sectional data were collected in 2016 from 1919 adults in 12 communities in Kenya and Uganda, to examine mobility (labour/non‐labour‐related travel), migration (changes of residence over geopolitical boundaries) and their associations with sexual behaviours (concurrent/higher risk partnerships), by region and sex. Multilevel mixed‐effects logistic regression models, stratified by sex and adjusted for clustering by community, were fitted to examine associations of mobility with higher‐risk behaviours, in past 2 years/past 6 months, controlling for key covariates.

**Results:**

The population was 45.8% male and 52.4% female, with mean age 38.7 (median 37, IQR: 17); 11.2% had migrated in the past 2 years. Migration varied by region (14.4% in Kenya, 11.5% in southwestern and 1.7% in eastern and Uganda) and sex (13.6% of men and 9.2% of women). Ten per cent reported labour‐related travel and 45.9% non‐labour‐related travel in past 6 months—and varied by region and sex: labour‐related mobility was more common in men (18.5%) than women (2.9%); non‐labour‐related mobility was more common in women (57.1%) than men (32.6%). In 2015 to 2016, 24.6% of men and 6.6% of women had concurrent sexual partnerships; in past 6 months, 21.6% of men and 5.4% of women had concurrent partnerships. Concurrency in 2015 to 2016 was more strongly associated with migration in women [aRR = 2.0, 95% CI(1.1 to 3.7)] than men [aRR = 1.5, 95% CI(1.0 to 2.2)]. Concurrency in past 6 months was more strongly associated with labour‐related mobility in women [aRR = 2.9, 95% CI(1.0 to 8.0)] than men [aRR = 1.8, 95% CI(1.2 to 2.5)], but with non‐labour‐related mobility in men [aRR = 2.2, 95% CI(1.5 to 3.4)].

**Conclusions:**

In rural eastern Africa, both longer‐distance/permanent, and localized/shorter‐term forms of mobility are associated with higher‐risk behaviours, and are highly gendered: the HIV risks associated with mobility are more pronounced for women. Gender‐specific interventions among mobile populations are needed to combat HIV in the region.

## 1 Introduction

Despite substantial progress in HIV treatment and prevention in sub‐Saharan Africa, highly mobile populations are proving challenging to engage in these gains. The significance of mobility in the HIV epidemic is clear: the early epidemic spread along major transportation routes 1, 2, 3, 4, mobile populations are faced with numerous individual‐ and structural‐level barriers to HIV diagnosis, treatment and prevention 5, 6, 7, 8, and may disproportionately contribute to onward transmission 9. However, there exist significant knowledge gaps concerning the complex forms of mobility emergent in sub‐Saharan Africa, their relationship to sexual behaviours, HIV acquisition and risk of onward transmission, as well as how sex modifies these associations. These knowledge gaps hinder the development and delivery of tailored interventions to successfully engage mobile persons in recent advances in HIV treatment and prevention.

Previous research into the relationships between mobility, sexual behaviours and HIV has been limited by measures that are broad, simplistic in their range and complexity, and has failed to take into account the significant heterogeneity of mobility by sex 10, 11. There are few data sources and metrics for population‐based estimates that are reflective of the complex forms of mobility emergent in sub‐Saharan Africa. This has precluded meta‐analytical summaries 11, 12, 13, and resulted in contradictory and inconclusive results in studies of the links between mobility, sexual behaviour and HIV 11.

The lack of adequate depth and breadth in current measures and dimensions of mobility is particularly problematic for research on HIV and mobility in sub‐Saharan Africa, where populations are highly mobile: sub‐Saharan Africa's intra‐continental emigration rate (65%) represents the largest south to south movement of people in the world 14. Levels of mobility in the region have risen dramatically in recent decades, in tandem with rapid social transformations, including the world's fastest rate of urbanization (from 23% urban in 1970 to 40% in 2005.^15^) Forms of mobility in sub‐Saharan Africa are diverse and complex relative to other regions: the paradigmatic rural‐to‐urban migration flow does not predominate in all settings 16; rather, counter‐urbanization 17 and circulation between rural areas, semi‐urban towns and the rural perimeters of cities are common 10, 18, 19, 20, 21, particularly among women. The temporary, localized forms of mobility, for example, frequent movements among several homesteads, are more common in women, but difficult to measure 21.

To overcome these previous research gaps, we leveraged an ongoing large scale, population‐level community cluster randomized trial of 334,512 persons in eastern Africa evaluating a test‐and‐treat strategy for HIV epidemic control, to embed a detailed sub‐study of mobility, sexual behaviours and HIV. In this sub‐study, we apply newly developed, high‐resolution measures of mobility and sexual relationship history to enhance our understanding of the behavioural and social pathways through which patterns of mobility may contribute to HIV acquisition and sustain onward transmission.

## 2 Methods

### 2.1 Study Setting

This mobility study is embedded within the *Sustainable East Africa Research in Community Health* (*SEARCH*) trial (NCT# 01864603), which seeks to test the effectiveness of the test and treat strategy for reducing population‐level HIV incidence 22, 23. SEARCH is a community cluster‐randomized controlled trial in 32 communities in one region in Kenya and two regions in Uganda. All communities received a census and population‐wide HIV testing at baseline. The SEARCH intervention used a hybrid mobile HIV testing approach, in which 2‐week, mobile, multi‐disease community health campaigns were followed up by home‐based testing of campaign non‐participants 22. In the 16 intervention communities, all individuals diagnosed as HIV‐seropositive were then referred for immediate anti‐retroviral therapy (ART) within a streamlined model of care that is patient‐centred and designed to reduce patient‐level barriers and maximize health system efficiency 24. The ongoing study of mobility within the trial (R01MH104132) is being conducted in 12 of the SEARCH trial communities. In the embedded mobility study, we are conducting original data collection using novel classifications and quantifications of mobility in individuals in the 12 communities in order to more fully understand the impact of mobility on sexual HIV risk behaviours, HIV prevalence and incidence, engagement in the HIV care continuum and components of the universal HIV testing and treatment intervention strategy. This analysis describes significant forms of mobility and their associations with sexual behaviours at study baseline.

### 2.2 Study sample

Our study enrolled 2750 adults aged 16 and older (with a consent rate of 98.3% to derive the cohort), of whom n = 480 simultaneously were enrolled in an embedded cohort of n = 240 couples. Individual‐level analyses were conducted with n = 2308 individuals who were not exclusively in the couples’ cohort (including n = 2270 who were exclusively in the individuals’ cohort and n = 38 who were in both cohorts). The analyses for this article were further restricted to n = 1919 sexually active individuals, defined as individuals reporting any sexual partnerships since January 2011. The dataset therefore excluded n = 389 individuals, among whom n = 76 reported not ever having had any sexual partners and n = 313 reported having been sexually active in the past but not since January 2011.

A multi‐level stratified random sampling design (region, intervention arm, HIV status, mobility status, gender) was used to select the sample of individuals from the census‐enumerated adult population of each of 12 SEARCH communities, composed of eight roughly equally sized groups of HIV‐positive and HIV‐negative, mobile and residentially stable men and women. The 12 communities, balanced by SEARCH study arm, were selected purposively to reflect underlying heterogeneity in forms of mobility across communities in SEARCH, and were composed of three communities each from the two regions of Uganda, along with three inland, and three Lake Victoria shoreline communities in Kenya. Baseline mobility for sampling purposes was defined on the basis of census data collected by the SEARCH trial on household presence in past 12 months (mobile = ‘away from household 6 months or more in past 12 months’, and/or “fewer than half of nights spent in household in past 4 months”). HIV‐positive individuals and mobile individuals were oversampled to achieve the desired sample size in each stratum. In communities with low total numbers of a stratum, all individuals were sampled in the stratum, a scenario that was common in communities with low HIV prevalence and low levels of mobility.

Sampling weights were generated based on the proportional representation of each of eight strata in the population census enumeration of the 12 communities. Sampling weights for stratum *i* in community $j\left(w_{i,j}\right)$ were calculated by dividing the expected number of individuals within each stratum (based on population proportions) by the observed number of individuals actually sampled in each stratum, as follows:

$$w_{i,j}=\frac{{\text{Expected}}_{i,j}}{{\text{Sampled}}_{i,j}}=\frac{\left(\frac{N_{i,j}}{N_{j}}\;\times \;n_{j}\right)}{n_{i,j}}$$

Where $N_{i,j}$ is the population size of stratum *i* in community *j*, $N_{j}$ is the total population size of community *j*, $n_{j}$ is the sample size of community *j*, and $n_{i,j}$ is the sample size of stratum *i* in community *j*.

### 2.3 Inclusion/exclusion criteria

Inclusion into the study was restricted to individuals in these 12 SEARCH communities, aged 16 and older, for whom baseline HIV serostatus and census‐defined mobility status was ascertained. As described in detail elsewhere 22, the SEARCH trial ascertained individuals’ HIV status via rapid, finger‐prick blood based HIV antibody testing and counselling using ministry of health test kits and testing algorithms.

### 2.4 Data collection

Mobility survey data were collected during one visit with study participants between February and November 2016. Data were collected in households or in another location preferred by the participant; research assistants, trained in research ethics and qualitative and survey interview techniques, conducted data collection in participants’ preferred local language. Research teams were gender‐balanced and gender‐matched to participants, to maximize rapport and reduce social desirability bias. Survey data were collected using programmed tablets and took about one and one‐half hours to complete; topics included demographics, migration histories, work and non‐work related travel in the past six months, and reasons for mobility. For HIV‐positive participants, additional data were collected about disclosure, experiences of stigma, and HIV care engagement.

### 2.5 Measurement of sexual behaviour

A Relationship History Calendar module, adapted from an instrument previously used in the region and shown to reduce social desirability bias to improve the reporting of sexual relationships and behaviour 25, was used to collect information about sexual behaviour and partnerships since January 2011. The calendar survey is a fold‐out grid with units of time in months and years noted across the top of the grid. The survey records information in monthly intervals rather than years, because many relationships survive for <1 year; we measured changes in relationship dimensions and behaviours over the course of each sexual relationship in the preceding approximately five years from the time of the survey (i.e. since January 2011). Use of the calendar survey enabled us to clearly identify sexual partner concurrency and its overlap with mobility over time.

The calendar was used to collect month‐by‐month data on partnerships, including relationship type and co‐residence and mobility of partners; durations; frequency of sex, contraceptive and condom use; and the exchange of gifts and/or money within each partnership. “High risk partner” refers to a sexual partnership with a casual partner, commercial sex worker or client, one night stand, or inherited partner/inheritor. Sexual partnership concurrency is occurrence of two or more sexual partnerships within any one month over a given time period. Occupation types, detailed in Table 1, are grouped into the categories “informal low risk”, “formal” and “informal high risk” on the basis of underlying HIV prevalence levels within those livelihood categories.

**Table 1:** Sociodemographic characteristics, by region and sex

Characteristic	Overall	Region				Sex		
		Kenya	Uganda E	Uganda SW	*p*	Men	Women	*p*
Weighted n	1898.1	1045	364	489.1		869.1	1029	
Region					NA			0.910
Kenya‐Western	1045.0 (55.1)	1045.0 (100.0)	0.0 (0.0)	0.0 (0.0)		479.4 (55.2)	565.5 (55.0)	
Uganda‐Eastern	364.0 (19.2)	0.0 (0.0)	364.0 (100.0)	0.0 (0.0)		167.2 (19.2)	196.8 (19.1)	
Uganda‐South Western	489.1 (25.8)	0.0 (0.0)	0.0 (0.0)	489.1 (100.0)		222.4 (25.6)	266.7 (25.9)	
10‐year age band					0.038			0.017
16 to 24	405.2 (21.3)	226.8 (21.7)	72.4 (19.9)	106.0 (21.7)		175.5 (20.2)	229.7 (22.3)	
25 to 34	616.6 (32.5)	320.5 (30.7)	133.1 (36.6)	162.9 (33.3)		250.6 (28.8)	366.0 (35.6)	
35 to 44	423.8 (22.3)	240.5 (23.0)	83.3 (22.9)	100.1 (20.5)		202.5 (23.3)	221.3 (21.5)	
45 to 54	207.6 (10.9)	105.7 (10.1)	53.7 (14.8)	48.2 (9.9)		93.4 (10.8)	114.2 (11.1)	
55 to 64	149.3 (7.9)	96.7 (9.3)	17.8 (4.9)	34.9 (7.1)		83.0 (9.6)	66.3 (6.4)	
65 and older	95.5 (5.0)	54.8 (5.2)	3.7 (1.0)	37.0 (7.6)		64.1 (7.4)	31.5 (3.1)	
Marital status					<0.001			0.022
Divorced, separated, widowed, missing	155.2 (8.2)	108.3 (10.4)	4.5 (1.2)	42.5 (8.7)		26.8 (3.1)	128.4 (12.5)	
Currently married	1543.8 (81.3)	815.7 (78.1)	345.2 (94.8)	382.9 (78.3)		734.1 (84.5)	809.7 (78.7)	
Currently single	199.1 (10.5)	121.0 (11.6)	14.3 (3.9)	63.8 (13.0)		108.2 (12.4)	90.9 (8.8)	
Education level					<0.001			0.007
No Schooling	193.8 (10.4)	41.2 (4.0)	68.4 (19.1)	84.2 (17.3)		61.0 (7.2)	132.8 (13.1)	
Primary/Secondary	1587.4 (85.0)	953.3 (93.2)	274.1 (76.5)	360.0 (74.1)		747.7 (88.0)	839.7 (82.6)	
Post‐secondary	85.3 (4.6)	27.8 (2.7)	16.0 (4.5)	41.4 (8.5)		41.1 (4.8)	44.1 (4.3)	
Household wealth: Poorest quantile	256.3 (13.5)	113.1 (10.8)	56.5 (15.5)	86.6 (17.7)	0.018	133.5 (15.4)	122.7 (11.9)	0.120
Occupation								
Informal sector (low risk)					0.239			<0.001
Farming/livestock	1037.5 (58.6)	461.0 (47.8)	302.8 (85.5)	273.6 (60.8)		441.2 (54.2)	596.3 (62.4)	
Student	22.1 (1.2)	22.1 (2.3)	0.0 (0.0)	0.0 (0.0)		21.2 (2.6)	0.9 (0.1)	
Construction/artisanal labour	65.8 (3.7)	41.6 (4.3)	0.0 (0.0)	24.2 (5.4)		65.1 (8.0)	0.7 (0.1)	
Shopkeeper/market vendor	272.4 (15.4)	160.0 (16.6)	23.7 (6.7)	88.8 (19.7)		87.7 (10.8)	184.8 (19.3)	
Household worker/housewife	5.7 (0.3)	5.0 (0.5)	0.0 (0.0)	0.6 (0.1)		3.4 (0.4)	2.3 (0.2)	
Informal sector (high risk)								
Fishing/fish trade	188.5 (10.7)	184.1 (19.1)	3.1 (0.9)	1.4 (0.3)		96.3 (11.8)	92.2 (9.7)	
Hotel/restaurant/bar worker	35.1 (2.0)	13.7 (1.4)	5.5 (1.6)	15.8 (3.5)		17.5 (2.2)	17.5 (1.8)	
Transport driver/tourism	41.4 (2.3)	30.4 (3.2)	0.0 (0.0)	11.0 (2.4)		39.4 (4.8)	2.1 (0.2)	
Formal sector								
Gov't/military/teacher/healthcare	95.7 (5.4)	42.1 (4.4)	19.1 (5.4)	34.5 (7.7)		38.9 (4.8)	56.8 (6.0)	
Factory worker/mining	4.7 (0.3)	4.6 (0.5)	0.0 (0.0)	0.1 (0.0)		3.1 (0.4)	1.5 (0.2)	

Weighted frequencies and column percentages shown.

Informal sector occupations categorized as “high risk” or “low risk” are defined on the basis of associations with underlying HIV prevalence in the SEARCH trial.

### 2.6 Measurement of mobility

The baseline mobility survey captured participants’ histories of migrations over their lifetime, by asking participants to tell us their birthplace and the names of places they lived (with county/district/nation recorded by the interviewer) along with their age at change of residence, in childhood through to the present. Reasons for moves were collected for the five most recent changes of residence. These data were used for measures of migration. Migration was defined as a movement of people across a specified geopolitical boundary for the purpose of establishing a new permanent residence. Migration between countries was classified as international; migration within countries, as internal migration. We used high‐resolution metrics in order to differentiate between shorter‐distance and longer‐distance internal migration: we recorded inter‐district/sub‐county (i.e. *across district*) as well as intra‐district/sub‐county (i.e. *within district*) changes of residence. Districts in Uganda, and sub‐counties in Kenya, are the geopolitical units that are most equivalent in population size and area.

After a set of questions about livelihoods, interviewers asked about mobility in the past six months. Participants were asked first about labour‐related mobility: *“The next questions are about the travelling that you do for business/to earn money. This includes travel to look for a job, and for farming/food production. Do you ever travel away from your home area for these purposes? (For this question, I'm talking about travel that requires your sleeping away from your main residence.)”* If participants answered yes, interviewers then probed to ask the names of ALL locations where participants travelled in past six months. Interviewers recorded the county/district of the location, the number of trips to the location, the length of nights spent on each trip to the location, where participants stayed, and the reasons for the trip(s) to the location. Interviewers then asked participants about non‐labour‐related mobility: “*Did you travel to any other places for other purposes (other than work) in the last six months?*” If participants answered yes, interviewers then probed to ask the names of ALL locations where participants travelled in past six months for reasons other than work, and recorded all subsequent information as described about frequency, duration and reasons. These data were used for measures of mobility: Mobility was defined as travel involving time spent away from primary places of residence, without any intention to change residence (locations and movements between multiple homes that are considered to be main residences are also recorded). This excluded commuting, as mobility is recorded only if the travel involved sleeping one or more nights away from primary residence(s). Labour‐related mobility was defined as travel “for business/to earn money”, including travel to look for a job, and for farming/food production. Non‐labour‐related mobility was defined as travel for all other purposes. Numbers of trips taken, and number of nights spent on each trip, by location, are collected for the previous 6 months before the visit date; a total number of nights was tallied over time periods by travel purpose.

### 2.7 Data analysis

Statistical analyses with individual‐level survey weights were used to describe population characteristics and test for their bivariate associations with sexual partnership concurrency and with higher risk partnerships over the period 2015 to 2016 and in the six months prior to the survey date, and implemented in the R statistical language version 3.2.2 using the survey package 26. Multilevel mixed‐effects logistic regression models, adjusted for clustering by community, were fitted using Stata statistical software (version 14.2) 27 to examine associations of measures of mobility with sexual behaviour measures, in past two years and in past six months respectively, controlling for key covariates (region, sex, age, marital status, occupation, and household wealth.) Subsequent models were fitted to test for interactions between sex and measures of mobility on sexual behaviour. We then estimated model‐adjusted risks 28 to compare predicted sexual behaviour outcomes: using Stata post estimation procedures (“margins, dydx”), we obtained average marginal effects of selected mobility metrics (dichotomous independent variables) from the fitted logistic regression models, summarized in Tables 6. The average marginal effect is the difference in the adjusted predictions for the two groups, or risk difference 28, from which adjusted marginal relative risks are obtained using the conversion method 29.

### 2.8 Ethical approval

Ethical approvals for this research were received from the University of California San Francisco Committee on Human Research (14‐15058), Ethical Review Committee of the Kenya Medical Research Institute (KEMRI/SERU/CMR/3052), Makerere University School of Medicine Research and Ethics Committee (2015‐040), and Uganda National Council for Science and Technology (HS 1834).

## 3 Results

### 3.1 Migration by Region and Sex

Tables 1 and 2 describe characteristics of the study population and their mobility and sexual behaviours by region and sex. Over half of the sample (50.6%) had at least one migration as an adult; nearly all of these were internal migrations (49.5%). A higher proportion of internal migrations occurred across district or sub‐county lines (38.3%), compared to intra‐district/sub‐county (23.6%) migrations. Over 20% had migrated in the past five years, 11.2% in the past two years, and 7.5% in the past year. International migration was rare, undertaken by men only, and almost exclusively intra‐continental (not shown). Levels of migration varied across regions, and were most prevalent in Kenya, with 27% having migrated in past five years, and 14.4% in past two  years. The only exception: a higher proportion of southwestern Ugandans (18%) than Kenyans (12.3%) undertook inter‐district/sub‐county migration in the previous five years (and in past two years, 11% *vs*. 4.6% respectively.) Higher proportions of men than women migrated in past five (22.4% *vs*. 18.2%) and two years (13.6% *vs*. 9.2%). However, women predominated in the more localized intra‐district/sub‐county migrations (25.6% *vs*. 21.2%) overall, with higher but non‐statistically significant differences in levels of intra‐district moves in the past five, two and one years.

**Table 2:** Mobility and sexual behaviour, by region and sex

Characteristic	Overall	Region				Sex		
		Kenya	Uganda E	Uganda SW	*p*	Men	Women	*p*
Weighted n	1898.1	1045	364	489.1		869.1	1029	
Any adult migration	959.9 (50.6)	733.9 (70.2)	41.6 (11.4)	184.4 (37.7)	<0.001	473.8 (54.5)	486.1 (47.2)	0.119
Any adult internal migration	938.7 (49.5)	716.2 (68.5)	41.6 (11.4)	180.9 (37.0)	<0.001	453.2 (52.1)	485.5 (47.2)	0.335
Intra‐district/sub‐county	447.8 (23.6)	445.2 (42.6)	0.2 (0.0)	2.4 (0.5)	<0.001	184.4 (21.2)	263.4 (25.6)	0.003
Inter‐district/sub‐county	727.1 (38.3)	505.6 (48.4)	41.5 (11.4)	179.9 (36.8)	<0.001	399.3 (45.9)	327.8 (31.9)	0.003
Any adult international migration	74.3 (3.9)	66.2 (3.5)	0.1 (0.0)	8.1 (0.4)	<0.001	61.2 (3.2)	13.1 (1.0)	<0.001
Past 5 years: Any migration	382.0 (20.1)	282.1 (27.0)	8.0 (2.2)	91.9 (18.8)	<0.001	194.5 (22.4)	187.4 (18.2)	0.028
Past 5 years: Any internal migration	377.5 (19.9)	280.3 (26.8)	8.0 (2.2)	89.2 (18.2)	<0.001	190.1 (21.9)	187.4 (18.2)	0.056
5‐year Intra‐District/Sub‐county	192.8 (10.2)	191.7 (18.3)	0.0 (0.0)	1.1 (0.2)	0.054	77.9 (9.0)	115.0 (11.2)	0.076
5‐year Inter‐District/Sub‐county	224.4 (11.8)	128.1 (12.3)	8.0 (2.2)	88.3 (18.0)	0.004	137.3 (15.8)	87.1 (8.5)	0.006
Past 5 years: international migration	8.2 (0.4)	4.1 (0.4)	0.0 (0.0)	4.2 (0.9)	0.471	8.2 (0.9)	0.0 (0.0)	0.032
Past 2 years: Any migration	212.9 (11.2)	150.4 (14.4)	6.2 (1.7)	56.3 (11.5)	0.004	117.8 (13.6)	95.1 (9.2)	0.002
Past 2 years: Any internal migration	211.5 (11.1)	150.4 (14.4)	6.2 (1.7)	54.9 (11.2)	0.003	116.3 (13.4)	95.1 (9.2)	0.003
2‐year Intra‐District/Sub‐county	106.9 (5.6)	105.9 (10.1)	0.0 (0.0)	1.0 (0.2)	0.104	42.7 (4.9)	64.2 (6.2)	0.138
2‐year Inter‐District/Sub‐county	107.9 (5.7)	47.8 (4.6)	6.2 (1.7)	53.9 (11.0)	0.001	76.4 (8.8)	31.5 (3.1)	0.009
Past 2 years: international migration	2.6 (0.1)	0.0 (0.0)	0.0 (0.0)	2.6 (0.5)	0.827	2.6 (0.3)	0.0 (0.0)	0.121
Past 1 year: Any migration	142.5 (7.5)	99.2 (9.5)	2.0 (0.5)	41.3 (8.4)	0.005	79.4 (9.1)	63.1 (6.1)	0.016
Past 1 year: Any internal migration	140.1 (7.4)	99.2 (9.5)	2.0 (0.5)	38.9 (8.0)	0.004	77.0 (8.9)	63.1 (6.1)	0.029
Past year intra‐district/sub‐county	67.9 (3.6)	67.0 (6.4)	0.0 (0.0)	1.0 (0.2)	0.120	27.6 (3.2)	40.3 (3.9)	0.409
Past year inter‐district/sub‐county	74.2 (3.9)	34.3 (3.3)	2.0 (0.5)	37.9 (7.8)	0.001	51.0 (5.9)	23.2 (2.3)	0.034
Past year international migration	2.4 (0.1)	0.0 (0.0)	0.0 (0.0)	2.4 (0.5)	0.835	2.4 (0.3)	0.0 (0.0)	0.135
Short‐term mobility								
Past 6 months mobility (≥1 nights away)								
Any labour‐related travel, past 6 month(%)	190.0 (10.0)	132.0 (12.6)	3.0 (0.8)	55.1 (11.3)	0.019	160.5 (18.5)	29.5 (2.9)	<0.001
No. labour‐related trips (mean (SD))	1.05 (9.27)	1.54 (12.00)	0.07 (1.39)	0.77 (5.02)	0.034	1.89 (12.25)	0.33 (5.39)	0.046
No. nights away, labour‐related travel (mean (SD))	3.08 (16.49)	3.13 (16.79)	0.50 (7.17)	4.94 (20.24)	0.020	5.42 (21.51)	1.06 (9.87)	0.008
Any non‐labour‐related travel, past 6 month (%)	871.0 (45.9)	612.9 (58.6)	20.8 (5.7)	237.3 (48.5)	<0.001	283.3 (32.6)	587.7 (57.1)	0.001
No. non‐work trips (mean (SD))	0.95 (1.80)	1.32 (2.08)	0.13 (0.67)	0.80 (1.51)	<0.001	0.59 (1.15)	1.27 (2.17)	0.007
No. nights away, non‐work travel (mean (SD))	4.29 (11.87)	5.47 (12.39)	0.89 (5.83)	4.37 (13.58)	<0.001	2.61 (9.01)	5.75 (13.73)	0.022
Past 1 month: Recent mobility (≥1 nights away)								
Any labour‐related travel, past 1 month (%)	108.3 (5.7)	80.3 (7.7)	3.0 (0.8)	25.0 (5.1)	0.015	91.2 (10.5)	17.1 (1.7)	<0.001
No. labour‐related trips (mean (SD))	0.21 (1.68)	0.31 (2.18)	0.02 (0.38)	0.13 (0.84)	0.035	0.38 (2.26)	0.06 (0.90)	0.040
No. nights away, labour‐related travel (mean (SD))	0.50 (3.06)	0.62 (3.38)	0.08 (1.16)	0.58 (3.30)	0.008	0.94 (4.12)	0.13 (1.57)	0.026
Any non‐labour‐related travel, past 1 month(%)	407.4 (21.5)	253.8 (24.3)	20.8 (5.7)	132.8 (27.2)	<0.001	127.1 (14.6)	280.3 (27.2)	0.001
No. non‐work trips (mean (SD))	0.27 (0.59)	0.31 (0.64)	0.09 (0.45)	0.31 (0.55)	0.002	0.19 (0.52)	0.34 (0.64)	0.011
No. nights away, non‐work travel (mean (SD))	0.94 (3.41)	0.91 (2.98)	0.58 (3.20)	1.28 (4.28)	0.134	0.83 (3.56)	1.04 (3.28)	0.244
Sexual behaviour								
Lifetime number of sexual partners (mean (SD))	5.2 (8.3)	6.5 (9.8)	3.0 (2.6)	4.2 (7.1)	<0.001	7.8 (11.1)	3.0 (3.6)	<0.001
Number of sexual partners, 2015 to 2016					0.005			<0.001
0 or 1	1605.3 (84.6)	832.9 (79.7)	340.1 (93.4)	432.3 (88.4)		645.2 (74.2)	960.1 (93.3)	
2	228.3 (12.0)	165.1 (15.8)	17.6 (4.8)	45.7 (9.3)		168.2 (19.4)	60.1 (5.8)	
3 or more	64.4 (3.4)	47.0 (4.5)	6.4 (1.8)	11.0 (2.3)		55.7 (6.4)	8.8 (0.9)	
Number of sexual partners, past 6 months					0.001			<0.001
0 or 1	1553.3 (85.7)	800.5 (80.8)	333.9 (94.1)	418.8 (89.4)		644.1 (76.5)	909.2 (93.6)	
2	206.7 (11.4)	149.5 (15.1)	17.1 (4.8)	40.1 (8.6)		150.8 (17.9)	56.0 (5.8)	
3 or more	53.3 (2.9)	40.2 (4.1)	3.7 (1.0)	9.4 (2.0)		47.3 (5.6)	5.9 (0.6)	
Sexual partnership concurrency								
Any concurrent sex partnerships, 2015 to 2016	284.6 (15.0)	203.9 (19.5)	24.0 (6.6)	56.7 (11.6)	<0.001	216.6 (24.9)	68.0 (6.6)	<0.001
Any concurrent sex partnerships, past 6 months	234.1 (12.9)	171.4 (17.3)	20.8 (5.9)	41.9 (8.9)	0.002	181.5 (21.6)	52.5 (5.4)	<0.001
Condom use, most recent month and partner					<0.001			
N/A (No sex)	203.3 (11.6)	87.4 (9.1)	19.6 (5.7)	96.3 (20.9)		75.7 (9.1)	127.6 (13.7)	0.061
Always	171.3 (9.8)	139.1 (14.6)	11.1 (3.2)	21.1 (4.6)		89.8 (10.8)	81.5 (8.8)	
Most of the time	40.2 (2.3)	26.9 (2.8)	11.9 (3.5)	1.3 (0.3)		30.7 (3.7)	9.5 (1.0)	
Sometimes	119.7 (6.8)	103.7 (10.9)	6.8 (2.0)	9.1 (2.0)		43.0 (5.2)	76.7 (8.3)	
Very rarely	11.0 (0.6)	1.9 (0.2)	0.1 (0.0)	9.0 (1.9)		5.6 (0.7)	5.4 (0.6)	
Never	1213.0 (69.0)	596.3 (62.4)	292.6 (85.5)	323.7 (70.3)		584.1 (70.5)	628.6 (67.6)	
Higher risk sexual partnerships								
Any higher risk sex partners, 2015 to 2016 (%)	225.9 (11.9)	174.8 (16.7)	0.3 (0.1)	50.8 (10.4)	<0.001	126.7 (14.6)	99.2 (9.6)	0.063
Any higher risk sex partners, past 6 month (%)	184.0 (9.7)	142.5 (13.6)	0.1 (0.0)	41.4 (8.5)	<0.001	98.9 (11.4)	85.1 (8.3)	0.199
HIV infection	260.1 (13.7)	217.9 (20.9)	8.9 (2.5)	33.3 (6.8)	<0.001	102.2 (11.8)	158.0 (15.4)	<0.001

Weighted frequencies and column percentages shown. Informal sector occupations categorized as “high risk” or “low risk” are defined on the basis of associations with underlying HIV prevalence in the SEARCH trial. “High risk partner”= casual partner, commercial sex worker or client, one night stand, or inherited partner/inheritor. Sexual partnership concurrency is occurrence of 2 or more sexual partnerships within any 1 month over a given time period.

### 3.2 Recent mobility by region and sex

Overall, 10% undertook any labour‐related travel, and 45.9% undertook travel for other reasons, in the previous six months. The mean number of labour‐related trips was higher than for non‐labour related trips, and the mean number of nights spent away was higher on labour‐related trips (16.5) than on non‐labour related (11.9). Levels of mobility of all types were significantly lower in eastern Uganda, comparable between southwestern Uganda and Kenya, but somewhat higher in Kenya for some forms of mobility. For example, 58.6% of Kenyans *versus* 48.5% of southwestern Ugandans undertook non‐labour related travel in the prior six months. Types of mobility by purpose varied significantly by sex: while 18.5% of men *versus* 2.9% of women undertook labour‐related travel (*p* ≤ 0.001), 57.1% of women *versus* 32.6% of men undertook travel for other purposes (*p *=* *0.001) in the past six months. The mean number of nights spent away for labour‐related travel was 21.5 for men *versus* 9.9 for women (*p *=* *0.008); in contrast, women spent 13.7 nights away and men spent 9.0 nights away on average on non‐labour‐related travel (*p *=* *0.022) in past six months.

### 3.3 Sex and regional differences in reasons for mobility

Participants were asked about the reasons for trips taken in the prior six months; these were significantly patterned by region and sex, as shown in Table 3. The predominant reasons men travelled for work were “artisanal labour (e.g. construction)” in Kenya (33.6%), and “market trading” in eastern (45.9%), and southwest Uganda (58.3%). The predominant reasons women travelled for work were “market trading” in Kenya (83.5%), and “looking for work” in southwest Uganda (77.3%) (in eastern Uganda fewer than five women travelled for livelihoods). Mobility in fishing communities figured predominantly in Kenya, where 27.9% of men and 11.1% of women travelled for livelihoods in the fish trade. The predominant reasons for non‐labour‐related mobility among men were for “attending funerals” in Kenya (42.4%), “holidays and visiting family” in south eastern Uganda (60.6%), and “care‐giving or care‐seeking” in southwest Uganda (58.5%). Among women, these were “attending funerals” in Kenya (50.1%) and eastern Uganda (85.7%), and “care‐giving or care‐seeking” in southwest Uganda (55.4%).

**Table 3:** Reasons for labour and non‐labour related mobility (among those reporting any mobility), by region and sex

Most common reported reason for travel	Men				Women			
	Kenya	Uganda E	Uganda SW	*p*	Kenya	Uganda E	Uganda SW	*p*
Weighted n (weighted %)	479.4	167.2	222.4		565.5	196.8	266.7	
Non‐labour‐related travel				<0.001				0.018
Holiday/Visiting family	9.6 (4.7)	7.4 (60.6)	15.2 (21.8)		35.8 (8.7)	0.6 (6.4)	16.0 (9.5)	
Funeral	85.5 (42.4)	0.8 (6.7)	9.0 (12.9)		205.9 (50.1)	7.4 (85.7)	36.4 (21.7)	
Care‐giving/Care‐seeking	68.5 (34.0)	0.8 (6.7)	40.7 (58.5)		107.2 (26.1)	0.6 (6.7)	93.1 (55.4)	
Other	33.4 (16.6)	3.2 (26.1)	0.5 (0.7)		60.7 (14.8)	0.1 (1.2)	18.3 (10.9)	
Schooling	4.6 (2.3)	0.0 (0.0)	4.2 (6.0)		1.7 (0.4)	0.0 (0.0)	4.0 (2.4)	
Labour‐related travel				0.070				NA
Artisanal labour (e.g. construction)	20.2 (33.6)	1.0 (33.8)	6.5 (19.6)					
Farming (own or others’ plots)	2.2 (3.7)	0.2 (8.3)	6.4 (19.1)		1.0 (5.1)	0.0 (NA)	1.0 (11.7)	
Fish trade	16.7 (27.9)	0.1 (3.7)	1.0 (2.9)		2.3 (11.1)	0.0 (NA)	0.0 (0.0)	
Looking for work	2.7 (4.5)	0.2 (8.3)	0.0 (0.0)		0.1 (0.3)	0.0 (NA)	6.8 (77.3)	
Market trading (incl. buying stock)	18.2 (30.3)	1.4 (45.9)	19.4 (58.3)		17.1 (83.5)	0.0 (NA)	1.0 (11.0)	

Weighted frequencies and column percentages shown.

### 3.4 Sexual risk behaviour

**Figure 1:**
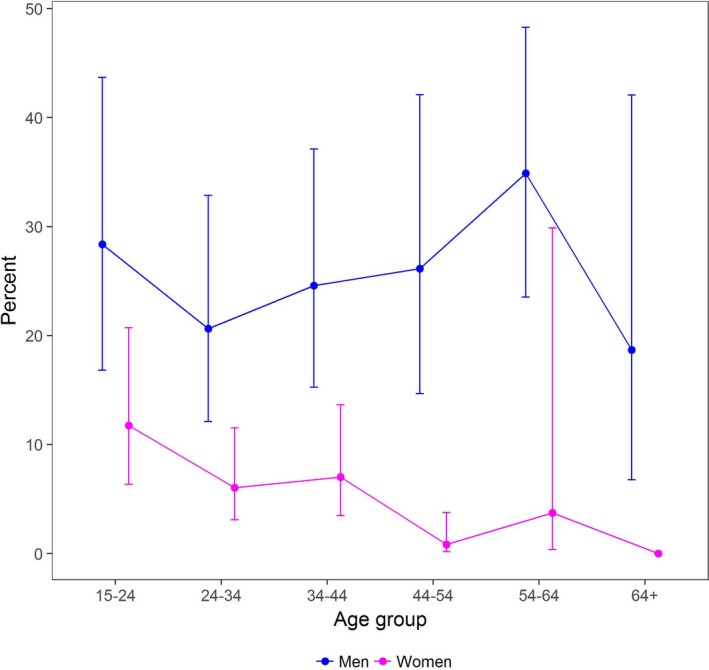
Prevalence of concurrent sexual partnerships in 2015 to 2016 among Sexually Active Adults, by 10‐year Age Band and Sex.

The mean number of lifetime sexual partners was 5.2 overall, significantly higher in Kenya (6.5) than in southwest (4.2) or eastern Uganda (3.0) (*p *≤ 0.001), and higher in men (7.8) than women (3.0) (*p *≤ 0.001) (Table 2). Similarly, measures of sexual partnership concurrency varied by region and sex. For example, 15% reported overlapping sexual partnerships in any month over the period 2015 to 2016; concurrency was highest in Kenya (19.5%), followed by southwestern (11.6%) and eastern Uganda (6.6%) (*p *≤ 0.001). A higher proportion of men (24.9%) than women (6.6%) reported sexual partnership concurrency (*p *≤ 0.001). Figure 1 shows the prevalence of concurrency by age and sex in 2015 to 2016. As shown, prevalence peaked in women in the youngest age band, declining thereafter; in contrast, prevalence of concurrency peaked in men aged 55 to 64. Condom use was rare, with only 9.8% always using condoms within a one‐month period. In 2015 to 2016, 11.9% had partnerships classified as higher risk (including casual partners and “one night stands”, commercial sex workers or clients, or inherited partners/inheritors), a proportion higher in Kenya (16.7%) than southwestern (10.4%) and eastern Uganda (0.1%) (*p *≤ 0.001); sex differences were non‐significant. Finally, prevalent HIV infection in the baseline year of the SEARCH study among this sample was 13.7% overall; 20.9% in Kenya, 6.8% in southwest and 2.5% in eastern Uganda (*p *≤* *0.001), and 15.4% in women *versus* 11.8% in men (*p *≤* *0.001).

### 3.5 Characteristics associated with higher risk sexual behaviours, by sex

The distribution of sex‐stratified population characteristics by concurrent sexual partnerships, and higher risk sexual partnerships, is shown for 2015 to 2016 (Table 4) and in the six months prior to survey (Table 5). Measures of mobility in the two tables differ in order to be temporally aligned with outcomes, with migration in 2015 to 2016 shown in Table 4, and past six‐month mobility shown in Table 5. Compared to those without concurrent sexual partners in 2015 to 2016, men and women who had any concurrent sexual partners were more likely to be Kenyan (*p *=* *0.006), HIV infected (*p *=* *0.004), and migrated in 2015 to 2016 (*p *=* *0.003). Concurrency also differed by age and marital status in women, and by occupation in men (Table 4); in addition, concurrency was associated with age and marital status in women, and with occupation in men. Women with concurrent partners in 2015 to 2016 were more likely to be younger (39.6%, *vs*. 21.1% of those without concurrency were ages 16 to 24) (*p *=* *0.035), and single (26.2%, compared to 7.6% of those without concurrent partners) (*p *=* *0.013).

**Table 4:** Characteristics associated with concurrent and higher risk sexual partnerships in 2015 to 2016, by sex

Characteristic	Women			Men			Women			Men		
	Concurrent sexual partnerships			Concurrent sexual partnerships			Higher risk partnerships			Higher risk partnerships		
	No	Yes	*p*	No	Yes	*p*	No	Yes	*p*	No	Yes	*p*
Weighted n	961.0	68.0		652.5	216.6		929.8	99.2		742.4	126.7	
Region	517.4 (53.8)		0.006			<0.001			<0.001			<0.001
Kenya ‐ Western		48.1 (70.7)		323.7 (49.6)	155.8 (71.9)		487.4 (52.4)	78.2 (78.8)		382.8 (51.6)	96.6 (76.3)	
Uganda ‐ Eastern	196.5 (20.4)	0.3 (0.4)		143.6 (22.0)	23.7 (10.9)		196.7 (21.2)	0.0 (0.0)		167.0 (22.5)	0.3 (0.2)	
Uganda ‐ South Western	247.0 (25.7)	19.6 (28.9)		185.3 (28.4)	37.1 (17.1)		245.7 (26.4)	21.0 (21.2)		192.6 (25.9)	29.8 (23.5)	
Age Band			0.035			0.240			0.145			<0.001
16 to 24	202.7 (21.1)	27.0 (39.6)		125.7 (19.3)	49.8 (23.0)		211.1 (22.7)	18.6 (18.8)		109.3 (14.7)	66.2 (52.3)	
25 to 34	343.9 (35.8)	22.1 (32.5)		198.9 (30.5)	51.7 (23.9)		338.1 (36.4)	27.9 (28.1)		219.0 (29.5)	31.5 (24.9)	
35 and older	414.4 (43.1)	18.9 (27.8)		327.9 (50.3)	115.1 (53.2)		380.6 (40.9)	52.7 (53.1)		414.1 (55.8)	29.0 (22.9)	
Marital Status			0.013			0.340			<0.001			<0.001
Divorced, Separated, Widowed, Missing	121.0 (12.6)	7.4 (10.9)		22.0 (3.4)	4.8 (2.2)		77.2 (8.3)	51.3 (51.7)		16.9 (2.3)	9.9 (7.8)	
Currently married	766.9 (79.8)	42.8 (62.9)		557.2 (85.4)	176.9 (81.7)		780.4 (83.9)	29.3 (29.5)		669.9 (90.2)	64.2 (50.7)	
Currently single	73.1 (7.6)	17.8 (26.2)		73.2 (11.2)	35.0 (16.1)		72.2 (7.8)	18.7 (18.8)		55.6 (7.5)	52.5 (41.5)	
Education level			0.287			0.653			0.475			0.007
No Schooling	129.8 (13.7)	3.0 (4.5)		49.4 (7.8)	11.6 (5.4)		121.5 (13.2)	11.3 (11.6)		59.3 (8.2)	1.7 (1.3)	
Primary/Secondary	775.5 (81.7)	64.2 (95.5)		556.4 (87.4)	191.2 (89.7)		753.3 (82.0)	86.4 (88.4)		626.3 (86.6)	121.4 (96.2)	
Post‐secondary	44.1 (4.6)	0.0 (0.0)		30.8 (4.8)	10.4 (4.9)		44.1 (4.8)	0.0 (0.0)		38.0 (5.2)	3.2 (2.5)	
Household wealth: Poorest quantile	112.2 (11.7)	10.6 (15.5)	0.533	106.7 (16.4)	26.8 (12.4)	0.334	105.0 (11.3)	17.7 (17.9)	0.112	108.7 (14.6)	24.9 (19.6)	0.479
Occupation			0.094			<0.001			0.254			<0.001
Informal sector: low risk	738.2 (76.8)	45.9 (67.5)		480.2 (73.6)	117.1 (54.1)		709.8 (76.3)	74.3 (74.9)		538.1 (72.5)	59.2 (46.7)	
Formal sector	56.5 (5.9)	1.9 (2.8)		32.9 (5.0)	9.1 (4.2)		55.6 (6.0)	2.7 (2.8)		38.5 (5.2)	3.5 (2.8)	
Informal sector: high risk	103.5 (10.8)	9.3 (13.6)		101.8 (15.6)	72.6 (33.5)		94.7 (10.2)	18.0 (18.1)		127.0 (17.1)	47.4 (37.4)	
HIV infection	139.8 (14.5)	18.2 (26.8)	0.004	65.5 (10.0)	36.7 (16.9)	0.001	122.9 (13.2)	35.1 (35.4)	0.005	84.1 (11.3)	18.1 (14.3)	0.394
Migration in 2015 to 2016: Any	74.7 (7.8)	20.4 (30.0)	0.003	73.0 (11.2)	44.8 (20.7)	0.011	81.7 (8.8)	13.4 (13.5)	0.334	80.8 (10.9)	37.0 (29.2)	0.004
Any internal migration	74.7 (7.8)	20.4 (30.0)	0.003	71.7 (11.0)	44.7 (20.6)	0.008	81.7 (8.8)	13.4 (13.5)	0.334	79.5 (10.7)	36.9 (29.1)	0.003
Intra‐District/Sub‐county	55.0 (5.7)	9.2 (13.5)	0.023	19.4 (3.0)	23.3 (10.8)	0.006	55.6 (6.0)	8.7 (8.7)	0.509	22.3 (3.0)	20.3 (16.0)	0.004
Inter‐District/Sub‐county	20.3 (2.1)	11.2 (16.5)	<0.001	53.4 (8.2)	23.0 (10.6)	0.324	26.3 (2.8)	5.2 (5.2)	0.356	59.3 (8.0)	17.1 (13.5)	0.027
Any international migration	NA	NA	NA	2.3 (0.4)	0.3 (0.1)	0.205	NA	NA	NA	2.3 (0.3)	0.3 (0.2)	0.665

Data are weighted; column percentages shown; missing data excluded for education level and occupation.

**Table 5:** Characteristics associated with concurrent and higher risk sexual partnerships in past 6 months, by sex

Characteristic	Women			Men			Women			Men		
	Concurrent sexual partnerships			Concurrent sexual partnerships			Higher risk partnerships			Higher risk partnerships		
	No	Yes	*p*	No	Yes	*p*	No	Yes	*p*	No	Yes	*p*
Weighted n	918.6	52.5		660.6	181.5		943.9	85.1		770.2	98.9	
Region			0.078			<0.001			0.001			<0.001
Kenya‐Western	490.6 (53.4)	37.6 (71.6)		328.3 (49.7)	133.8 (73.7)		498.1 (52.8)	67.4 (79.2)		404.4 (52.5)	75.1 (75.9)	
Uganda‐Eastern	193.4 (21.0)	0.1 (0.1)		140.6 (21.3)	20.7 (11.4)		196.7 (20.8)	0.0 (0.0)		167.2 (21.7)	0.0 (0.0)	
Uganda‐South Western	234.6 (25.5)	14.9 (28.3)		191.7 (29.0)	27.0 (14.9)		249.0 (26.4)	17.6 (20.7)		198.6 (25.8)	23.8 (24.0)	
Age Band			0.652			0.008			0.081			<0.001
16 to 24	207.9 (22.6)	15.7 (29.9)		112.3 (17.0)	45.6 (25.1)		213.3 (22.6)	16.4 (19.3)		118.3 (15.4)	57.2 (57.8)	
25 to 34	337.1 (36.7)	18.9 (36.1)		212.0 (32.1)	34.9 (19.2)		343.4 (36.4)	22.6 (26.6)		231.3 (30.0)	19.2 (19.4)	
35 and older	373.6 (40.7)	17.9 (34.0)		336.3 (50.9)	101.0 (55.6)		387.2 (41.0)	46.1 (54.2)		420.6 (54.6)	22.5 (22.7)	
Marital status			0.027			0.005			<0.001			<0.001
Divorced, Separated, Widowed, Missing	78.0 (8.5)	5.6 (10.7)		21.4 (3.2)	1.6 (0.9)		84.4 (8.9)	44.1 (51.8)		18.8 (2.4)	8.0 (8.1)	
Currently married	775.6 (84.4)	33.4 (63.7)		579.5 (87.7)	149.8 (82.5)		783.4 (83.0)	26.3 (30.9)		694.6 (90.2)	39.5 (39.9)	
Currently single	65.0 (7.1)	13.5 (25.7)		59.6 (9.0)	30.1 (16.6)		76.1 (8.1)	14.8 (17.4)		56.8 (7.4)	51.4 (51.9)	
Education level			0.452			0.623			0.421			0.002
No Schooling	113.6 (12.5)	3.0 (5.8)		50.8 (7.9)	9.5 (5.3)		124.9 (13.4)	8.0 (9.5)		60.0 (8.0)	1.0 (1.0)	
Primary/Secondary	751.8 (82.9)	48.7 (94.2)		563.6 (87.3)	158.6 (89.0)		764.0 (81.9)	75.7 (90.5)		653.2 (86.9)	94.5 (96.1)	
Post‐secondary	41.7 (4.6)	0.0 (0.0)		30.9 (4.8)	10.1 (5.7)		44.1 (4.7)	0.0 (0.0)		38.2 (5.1)	2.9 (3.0)	
Household wealth: Poorest quantile	108.9 (11.9)	8.7 (16.6)	0.505	109.3 (16.5)	20.3 (11.2)	0.218	105.3 (11.2)	17.4 (20.5)	0.061	114.0 (14.8)	19.5 (19.7)	0.562
Occupation			0.312			0.006			0.205			0.004
Informal sector: low risk	706.6 (76.9)	38.6 (73.4)		484.7 (73.4)	105.0 (57.8)		722.3 (76.5)	61.8 (72.5)		553.1 (71.8)	44.2 (44.7)	
Formal sector	54.6 (5.9)	1.0 (1.8)		30.5 (4.6)	8.5 (4.7)		55.6 (5.9)	2.7 (3.2)		39.1 (5.1)	3.0 (3.0)	
Informal sector: high risk	98.7 (10.7)	6.6 (12.5)		112.1 (17.0)	50.2 (27.7)		95.7 (10.1)	17.1 (20.1)		139.0 (18.1)	35.4 (35.8)	
Missing	58.6 (6.4)	6.4 (12.2)		33.4 (5.1)	17.8 (9.8)		70.3 (7.4)	3.6 (4.2)		38.9 (5.1)	16.4 (16.6)	
HIV infection	129.7 (14.1)	12.1 (23.0)	0.065	67.0 (10.1)	32.3 (17.8)	0.001	128.4 (13.6)	29.6 (34.8)	0.007	89.0 (11.6)	13.2 (13.3)	0.572
Mobility in Past 6 months												
Any labour‐related travel, past 6 months	22.8 (2.5)	4.7 (9.0)	0.103	110.7 (16.7)	45.7 (25.2)	0.018	21.3 (2.3)	8.3 (9.7)	0.015	140.3 (18.2)	20.2 (20.4)	0.742
No. nights away, labour‐related travel	0.79 (8.68)	5.72 (21.66)	0.326	4.33 (17.99)	9.37 (30.88)	0.036	0.50 (5.96)	6.91 (26.68)	0.131	5.03 (20.64)	8.34 (27.12)	0.387
Any non‐labour‐related travel, past 6 months	512.2 (55.8)	40.4 (77.0)	0.022	198.9 (30.1)	81.3 (44.8)	0.024	526.7 (55.8)	61.0 (71.7)	0.096	226.1 (29.4)	57.2 (57.8)	0.006
No. nights away, non‐work travel	1.24 (2.19)	1.76 (1.70)	0.086	0.53 (1.09)	0.80 (1.33)	0.013	1.24 (2.21)	1.53 (1.67)	0.325	0.53 (1.10)	1.02 (1.43)	0.023
Sexual behaviour, Past 6 months												
Any concurrent sex partners, past 6 months							23.5 (2.6)	29.1 (34.1)	<0.001	120.2 (16.2)	61.4 (62.0)	<0.001
Any high risk sex partners, past 6 month	56.1 (6.1)	29.1 (55.3)	<0.001	37.5 (5.7)	61.4 (33.8)	<0.001						
Number of sexual partners, past 6 months			<0.001			<0.001			<0.001			<0.001
0 or 1	909.2 (99.0)	0.0 (0.0)		644.1 (97.5)	0.0 (0.0)		853.3 (96.3)	55.9 (65.7)		608.8 (81.9)	35.3 (35.7)	
2	9.3 (1.0)	46.7 (88.9)		16.6 (2.5)	134.2 (73.9)		29.0 (3.3)	26.9 (31.6)		115.9 (15.6)	34.8 (35.2)	
3 or more	0.1 (0.0)	5.8 (11.1)		0.0 (0.0)	47.3 (26.1)		3.7 (0.4)	2.3 (2.7)		18.6 (2.5)	28.8 (29.1)	

Data are weighted; column percentages shown; missing data excluded for education level and occupation.

Concurrency was disproportionately higher among those in ‘informal high risk’ occupations: 33.5% of men and 13.6% of women with concurrent partners had these occupations (*vs*. 15.6% of men and 10.8% of women without concurrent partners in these occupations); these associations were highly significant in men but only approached significance in women (*p *=* *0.090). Concurrency was more prevalent than not among women who were fish traders (14.1% *vs*. 9.4%), market traders (26.5% *vs*. 18.9%), and hotel/restaurant/bar workers (2.2% *vs*. 1.8%); and among men who were fishermen (19.4% *vs*. 9.4%), transport drivers (8.3% *vs*. 3.7%) and hotel/restaurant/bar workers (4.8% *vs*. 1.3%) (not shown).

**Figure 2:**
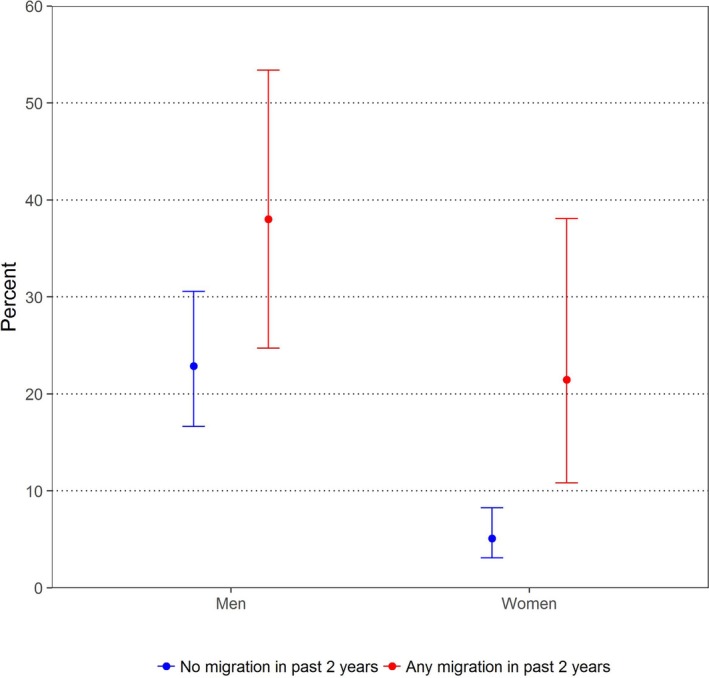
Prevalence of Sexual Partnership Concurrency in 2015 to 2016, by Sex and Migration in 2015 to 2016.

Participation migration in 2015 to 2016 was elevated in those with concurrent partners in 2015 to 2016. Among women, 30% of those in concurrent partnerships reported having migrated in 2015 to 2016 (*vs*. 7.8% not in such partnerships)(*p *=* *0.003), and the respective differences were especially pronounced for migrations across district/sub‐county boundaries (16.5% *vs*. 2.1%) (*p *≤* *0.001). In men, 20.6% of those in concurrent partnerships (*vs*. 11.2% not in such partnerships) migrated in 2015 to 2016 (*p *=* *0.011); there was no statistically significant association between concurrency and inter‐district/sub‐county migration. Figure 2 illustrates the associations of past migration in 2015 to 2016 with prevalence of sexual concurrency in the same time period for both men and women.

In women, engagement in higher risk sexual partnerships over 2015 and 2016 was significantly associated only with Kenyan residence and with marital status: women with higher risk partners were disproportionately divorced/separated/widowed (51.7% *vs*. 8.3%) or single (18.8% *vs*. 7.8%)(*p *≤* *0.001). In men, having higher risk sexual partners was associated with Kenyan residence, younger age group, middle education level, informal high‐risk occupation, and past two‐year internal migrations of all types. Over 29% of men who had higher risk partners from 2015 to 2016 (*vs*. 10.9% without such partners) had migrated during the same time period (*p *=* *0.004).

**Figure 3:**
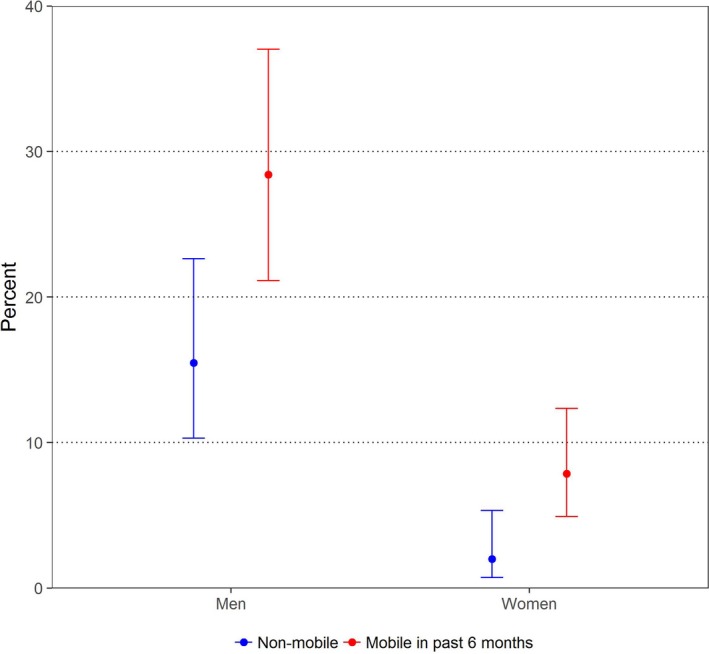
Prevalence of Sexual Partnership Concurrency in past 6 months, by Sex and Mobility.

In the six‐month period prior to survey (Table 5), concurrency was disproportionately more prevalent among women not currently married (*p *=* *0.027), and women who had undertaken any non‐labour‐related travel (77% in concurrent relationships *vs*. 55.8% not in such relationships) (*p *=* *0.022). In men concurrency was disproportionately more prevalent among those who were Kenyan (*p *≤* *0.001), older (*p *=* *0.008), not currently married (*p *=* *0.005), in informal sector employment (*p *=* *0.006), and who had undertaken past six‐month mobility of all types (for example, 25.2% in concurrent relationships *vs*. 16.7% not in such relationships had travelled for work) (*p *=* *0.018). In women, having higher risk sexual partners in the past six months was more prevalent among those who were Kenyan (*p *=* *0.001), unmarried (*p *≤ 0.003), and travelled for work (9.7% with higher risk partners *vs*. 2.3% without such partners) (*p *=* *0.015). For men, higher risk partnerships were more prevalent among men who were younger and unmarried (*p *≤* *0.001), and in informal sector employment (*p *=* *0.004), and who travelled for non‐labour‐related reasons (57.8% with higher risk partners *vs*. 29.4% without such partners) (*p *=* *0.006). Figure 3 illustrates the association of any past six‐month mobility with prevalence of sexual concurrency in the past six months for both men and women. A Supplemental Figure S1, displays the same associations, but by the types of past six month mobility.

### 3.6 Multivariate relationships between mobility and higher risk sexual behaviours

Table 6 shows summary mobility metrics (with model‐adjusted relative risks of outcomes) from multilevel mixed effects logistic regression models fitted on four outcomes, sequentially: concurrency in 2015 to 2016, and in the past six months, and higher risk sexual partnerships in 2015 to 2016, and in the past six months. Models were adjusted for clustering at the community level, controlled for region, age group, marital status, occupation, and HIV status, and stratified by sex (full output of the models is shown in supplementary Tables S3, S4.) Having undertaken any migration in 2015 to 2016 was associated with concurrency in 2015 to 2016 among women [aRR = 1.99, 95% CI (1.08 to 3.68)] and men [aRR = 1.47, 95% CI (1.00 to 2.16)]. Intra‐district/sub‐country migration in 2015 to 2016 was not associated with concurrency in 2015 to 2016 in either men or women, but inter‐district migration was, in women only [aRR = 2.93, 95% CI (1.18 to 7.28)]. Having undertaken any labour‐related travel in the past six months was associated with concurrency in the past six months in women [aRR = 2.86, 95% CI (1.03 to 7.96)] and men [aRR = 1.75, 95% CI (1.24 to 2.49)]. Non‐labour‐related travel in the past six months was associated with concurrency in the past six months in men only [aRR = 1.40, 95% CI (1.01 to 1.94)]. Migration in 2015 to 2016 was not associated with having higher‐risk partnerships in 2015 to 2016, in adjusted models, but there were associations between mobility and higher risk partnerships in the past six months: having higher risk partners was associated with labour‐related travel in women [aRR = 2.32, 95% CI (1.01 to 5.33)] and non‐labour‐related travel in men [aRR = 2.33, 95% CI (1.45 to 3.43)]. Tests for interactions between sex and key mobility metrics yielded null findings, but were suggestive; the coefficient for the interaction between sex and migration for the prediction of concurrency in 2015 to 2016 was non‐significant at *p *=* *0.07.

**Table 6:** Associations of measures of mobility with concurrent and higher risk sexual partnerships over a 2‐year period (2015 to 2016) and in past 6 months

Mobility metric	Adjusted risk ratio, any concurrent sexual partnership, 2015 to 2016												Adjusted risk ratio, any higher risk sexual partnership, 2015 to 2016											
	All				Women				Men				All				Women				Men			
	aRR	*p*	95% CI		aRR	*p*	95% CI		aRR	p	95% CI		aRR	*p*	95% CI		aRR	*p*	95% CI		aRR	*p*	95% CI	
Past 2 years: any migration	1.58	0.006	1.14	2.18	1.99	0.027	1.08	3.68	1.47	0.049	1.00	2.16	1.32	0.119	0.93	1.88	1.07	0.835	0.56	2.03	1.53	0.061	0.98	2.40
Past 2 year internal migration	1.60	0.005	1.15	2.23	1.99	0.027	1.08	3.68	1.50	0.042	1.01	2.21												
Past 2 year inter‐district/sub‐co. migration	1.63	0.020	1.08	2.45	2.93	0.021	1.18	7.28	1.43	0.126	0.90	2.25												
Past 2 year intra‐district/sub‐co. migration	1.38	0.174	2.95	11.63	1.35	0.443	0.63	2.92	1.43	0.228	0.80	2.56												

Mobility metric	Adjusted risk ratio, any concurrent sexual partnership, past 6 months												Adjusted risk ratio, any higher risk sexual partnership, past 6 months											
	All				Women				Men				All				Women				Men			
	aRR	*p*	95% CI		aRR	*p*	95% CI		aRR	p	95% CI		aRR	*p*	95% CI		aRR	*p*	95% CI		aRR	*p*	95% CI	
Any labour‐related travel, past 6 month	1.85	0.000	1.33	2.57	2.86	0.044	1.03	7.96	1.75	0.002	1.24	2.49	1.23	0.308	0.82	1.85	2.32	0.049	1.01	5.33	1.21	0.430	0.75	1.95
Any non‐labour‐related travel, past 6 month	1.40	0.022	1.05	1.87	1.22	0.531	0.65	2.30	1.40	0.043	1.01	1.94	1.91	0.000	1.38	2.65	1.45	0.157	0.87	2.43	2.23	0.000	1.45	3.43
No. nights away, labour‐related travel	1.01	0.000	1.00	1.01	1.01	0.195	1.00	1.02	1.01	0.001	1.00	1.01	1.00	0.303	1.00	1.01	1.01	0.017	1.00	1.03	1.00	0.763	0.99	1.01
No. nights away, non‐work travel	1.01	0.036	1.00	1.02	1.01	0.100	1.00	1.02	1.01	0.282	0.99	1.02	1.00	0.623	0.99	1.01	1.00	0.815	0.99	1.02	1.00	0.747	0.99	1.02

Results in Table 6 are from multilevel mixed‐effects logistic regression models adjusted for clustering at community level and controlling for region, age group, marital status, occupation, and HIV status. aRRs calculated using a linear prediction of average adjusted individual marginal effects of selected mobility metrics (1 vs. 0) from models. Models of mobility metrics beyond any past 2 year migration not fitted for prediction of higher risk sexual partnerships because of null findings in the initial model.

## 4 Discussion

The findings of this study in rural populations in Kenya and Uganda underscore the highly gendered nature of mobility and its influence on higher‐risk sexual behaviour. There was significant heterogeneity in mobility (movements to and from households of primary residence) and migration (moves to change residence, across geopolitical boundaries) across regions in Kenya and Uganda, which correlated with heterogeneous levels of risk behaviour and HIV prevalence observed across the regions. As we 10 and others 30, 31, 32 have previously documented in rural settings in South Africa, Uganda and Kenya, while men overall are more mobile than women, associations between population mobility and sexual risk behaviour were observed to be stronger in women compared to men. Levels of concurrency are elevated among both men and women who migrated or travelled compared to their more residentially stable counterparts, but the differences in levels of risk behaviour were greater for women than for men. For example, even though lower proportions of women (9.2%) than men (13.6%) migrated in the past 2 years, that migration was associated with a markedly higher risk of sexual partnership concurrency among women (aRR 2.0), than in men (aRR 1.5). Similarly, women were less likely (2.9%) than men (18.5%) to travel away from home for labour‐related purposes in the prior six months, but women who did had a higher risk of sexual partnership concurrency in that period (aRR 2.9) than did men who travelled for those reasons (aRR 1.8). Our findings support evidence that the pathway linking mobility to HIV acquisition and transmission in sub‐Saharan Africa is through higher risk sexual behaviour, and that these relationships are especially pronounced in women; longitudinal data will be required to confirm these findings.

Our study builds on a growing theoretical framework for empirical research on mobility and HIV (including key work by Cassels et al.^33^), by including high‐resolution measures inclusive of both men's and women's forms of and motivations for mobility. A unique contribution of this study to that literature is the finding that sex differences in the behavioural HIV risks associated with mobility are influenced by the underlying purposes of that mobility. The findings also show that not all markers of higher risk sexual behaviour are equivalent: whilst concurrency was associated with multiple measures of migration and mobility among both men and women, having higher risk partnerships was associated with certain types of mobility that differed by sex: having higher risk partners was associated with labour‐related travel in women (aRR = 2.3) and non‐labour‐related travel in men (aRR = 2.3). Labour‐related mobility was associated with concurrent partnerships in both women (aRR 2.9) and men (aRR 1.8), yet it conferred risk of higher risk partnerships only in women (aRR 2.3); that mobility was conducted primarily for livelihoods associated with market trading and the fish trade in women in Kenya. For men, non‐labour‐related mobility was associated with having concurrent (aRR 1.4) and higher risk partnerships (aRR 2.2); and that mobility was undertaken for more varied reasons including attending funerals, in Kenya, and care‐seeking or care‐giving or travelling for holidays to visit family in Uganda. For both men and women, higher risk sexual behaviour co‐occurred with the type of mobility that was less common for their sex (18.5% of men *vs*. 2.9% of women travelled for labour‐related purposes; 57.1% of women *vs*. 32.6% of men travelled for other purposes in the past six months).

Our prior research has documented the ways in which women's labour‐related mobility in Kenya—the livelihoods that women engage in that require their mobility—often involve sexual behaviour that can increase HIV acquisition and onward transmission risks 19. The circumstances that drive migration (e.g. widowhood) may increase HIV risk at the community of origin, and social contexts at destinations and transit points facilitate multiple and higher risk sexual partnerships. We found higher HIV prevalence among female market traders (25.6% in 2013) relative to a comparable population of women of reproductive age from a household survey in Kisumu in western Kenya (15.3% in 2013) 34. Our qualitative research has revealed that the more mobile market traders (in contrast to those who did not travel) often supplemented their low and sporadic earnings from market trading with transactional sex; the practice was common enough that the phrase “she mixes her business” was used, we were told, to describe this practice. Women's travel away from home communities, and away from the social monitoring of their sexual behaviour that occurs within kinship networks (especially the “prying eyes” of mothers‐in‐law with whom women often reside), facilitated opportunities for such exchanges 19.

That men's labour‐related mobility was not associated with higher risk sexual behaviour reflects the social reality that for most men in our study communities, higher risk sexual behaviour is not embedded within livelihood strategies as it is for many women. Men's non‐labour‐related mobility, often associated with travel that is highly social and even familial (attending funerals, seeking care, visiting family), was associated with higher risk behaviour and this perhaps reflects the greater cultural acceptance of men's extramarital sexual behaviour. Prior research from Tanzania found that men living apart from their wives did not report more extramarital sex than men who co‐resided with their wives, but the opposite was true for women, among whom those living apart reported extramarital sex more often 35. Normative masculinities in these settings are such that men's extramarital affairs, while often leading to marital strife, are common and even valorised 36. In Kenya, funerals are multi‐day gatherings of friends and family at the home of the deceased, accompanied by music and dancing; these are also occasions in which casual, transactional unprotected sex is common 37.

The findings presented here also confirm our prior research documenting high levels of mobility and HIV risk in communities on the shores of Lake Victoria: fishermen follow the fish over great distances, and female fish traders, who buy, process, transport and retail the fish in local markets, are also highly mobile 38, 39. Moreover, a lakeside transactional sexual economy known as “sex‐for‐fish”, or “*jaboya*”, contributes to the continued spread of HIV in these communities 38, 40 and those effects are likely to be amplified through women's mobility necessary to access fish and to sell in local and distant markets.

Reduced to its simplest message, this study's findings show that in predominantly rural eastern African settings, both migration and also localized short‐term mobility—whether labour‐related travel, or travel for other purposes—are associated with higher risk sexual behaviour, and that types of mobility and their relationship to sexual behaviours are strongly influenced by gender.

Our findings are of significant importance for understanding the context of the larger test and treat intervention trial in which this study is embedded. SEARCH demonstrated the effectiveness of its model for high HIV “cascade coverage”, and demonstrated an increase in virologic suppression rates from 45% to 81% among intervention communities 23. As of the time of this publication, measurement of the impact of the “test and treat” intervention on community‐level HIV incidence is underway. The impact of mobility on the efficacy of “test and treat” is not yet known, but mathematical modelling of its potential effect has suggested that the movement of individuals in and out of communities and care systems may substantially attenuate the gains of test and treat 41 and other HIV prevention interventions. We have recently argued 9 that even strategies that successfully meet or exceed the 90‐90‐90 targets will leave up to 27% of people living with HIV/AIDS virally non‐suppressed, and that the sexual behaviour, mobility, and network connectedness of this “missing 27%” must be better characterized to fully evaluate the effectiveness of and barriers to the universal test and treat strategy. Moreover, even demonstrated effectiveness in one setting does not ensure that these strategies will work everywhere. Understanding the potential effects of mobility on sexual behaviours and engagement in HIV prevention and treatment interventions could be critical for settings where mobility is common and on the rise.

Our study has several limitations. First, the data are cross‐sectional, serving as a baseline characterization of a cohort being followed longitudinally. The direction of causality between mobility and sexual risk cannot therefore be conclusively determined. Higher risk sexual behaviour may precede mobility, follow it as a consequence, or an unmeasured “predisposition to risk‐taking” may underlie both mobility and risk behaviour. Second, we cannot fully ascertain the pathways between mobility, risk and HIV prevalence in the region. That being said, the strength and consistency of relationships seen in this study can provide significant evidence in support of hypotheses that can be longitudinally investigated and confirmed in future study. There are strong associations between mobility and baseline HIV infection observed in this and other settings and plausible causal pathways leading from mobility to infection and in the other directions can be made. Prior research has shown that marital instability following HIV infection can lead to migration 42, and that people living with HIV are known to move to seek better care 43. Lastly, data for understanding the dynamics of mobility and sexual risk behaviour at the couple level was not available for this analysis. Future dyadic analyses, and longitudinal data, will be necessary to answer important research questions concerning relationship dynamics and links between mobility and HIV in these settings.

## 5 Conclusions

In rural eastern African settings, both long‐distance and permanent, and localized, short‐term forms of mobility were associated with higher‐risk sexual behaviour. Types of mobility, and their influence on sexual behaviour were found to be highly gendered and while overall men are more mobile than women, the behavioural risks associated with mobility are more pronounced for women than for men. Taken together, our research has significant implications for the design and evaluation of HIV prevention and treatment interventions that need to address the strong links between mobility and sexual HIV risk behaviours. Moreover, this work highlights the need for gender‐specific interventions among mobile populations to combat the ongoing HIV epidemic in sub‐Saharan Africa.

## Funding

Research reported in this article was supported by the National Institutes of Health, NIMH under award number R01MH104132 (Camlin, Mobility in SEARCH), and NIAID under award number U01AI099959 (Havlir, SEARCH), and in part by the President's Emergency Plan for AIDS Relief, Bill and Melinda Gates Foundation, and Gilead Sciences.

## Authors’ contributions

CSC, TBN and EDC designed the study, with contributions from MG, CRC, MRK and EAB. CSC and AA conducted the statistical analysis; TBN and MG contributed to data analysis and interpretation. CSC took primary responsibility and EDC and AA secondary responsibility for writing the manuscript. MG, PE and IM oversaw data collection and curation. All authors were involved in review, critiquing and editing of the manuscript.

## Competing interests

The authors have no competing interests to declare.

## Supporting information


**Table S1.** Characteristics Associated with Number of Sexual Partnerships in 2015 to 2016, by Sex.
**Table S2.** Characteristics Associated with Number of Sexual Partnerships in Past 6 months, by Sex.
**Table S3.** Associations of Measures of Mobility with Concurrent Sexual Partnerships Over a 2‐year Period (2015 to 2016) [Full model output].
**Table S4.** Associations of Measures of Mobility with Higher Risk Sexual Partnerships Over a 2‐Year Period (2015 to 2016) [Full model output].

**Figure S1.**Prevalence of Sexual Partnership Concurrency in Past 6 Months, by Sex and Mobility in Past 6 Months, by Type of Mobility.
