# Supplementary material for: Population mobility associated with higher risk sexual behaviour in eastern African communities participating in a Universal Testing and Treatment trial
Source: J Int AIDS Soc. 2018 Jul 19;21(Suppl Suppl 4):e25115. doi: 10.1002/jia2.25115 (PMC6053476; doi:10.1002/jia2.25115)
Supplement: Supplementary file 1 — Table S1. Characteristics Associated with Number of Sexual Partnerships in 2015 to 2016, by Sex. Table S2. Characteristics Associated with Number of Sexual Partnerships in Past 6 months, by Sex. Table S3. Associations of Measures of Mobility with Concurrent Sexual Partnerships Over a 2‐year Period (2015 to 2016) [Full model output]. Table S4. Associations of Measures of Mobility with Higher Risk Sexual Partnerships Over a 2‐Year Period (2015 to 2016) [Full model output]. [file JIA2-21-e25115-s001.docx]

**SUPPLEMENTAL TABLES**

**Table A.1: Characteristics Associated with Number of Sexual Partnerships in 2015-16, by Sex**

|  | **Women** | | | | **Men** | | | |
| --- | --- | --- | --- | --- | --- | --- | --- | --- |
|  | **Number of sexual partners in 2015-16** | | | | **Number of sexual partners in 2015-16** | | | |
| **Characteristic** | **0 or 1** | **2** | **3 or more** | ***p*** | **0 or 1** | **2** | **3 or more** | ***p*** |
| Weighted n | 960.1 | 60.1 | 8.8 |  | 645.2 | 168.2 | 55.7 |  |
| **Region** |  |  |  | 0.150 |  |  |  | 0.008 |
| Kenya - Western | 516.6 (53.8) | 43.8 (72.9) | 5.2 (58.9) |  | 316.4 (49.0) | 121.2 (72.1) | 41.8 (75.2) |  |
| Uganda - Eastern | 196.5 (20.5) | 0.3 (0.5) | 0.0 (0.0) |  | 143.6 (22.2) | 17.3 (10.3) | 6.4 (11.5) |  |
| Uganda - South Western | 247.0 (25.7) | 16.0 (26.7) | 3.6 (41.1) |  | 185.3 (28.7) | 29.7 (17.6) | 7.4 (13.4) |  |
| **Age Band** |  |  |  | 0.063 |  |  |  | 0.524 |
| 16-24 | 202.7 (21.1) | 22.3 (37.1) | 4.6 (53.0) |  | 121.6 (18.8) | 39.9 (23.7) | 14.0 (25.2) |  |
| 25-34 | 343.0 (35.7) | 22.6 (37.5) | 0.5 (5.2) |  | 196.2 (30.4) | 39.9 (23.7) | 14.5 (26.1) |  |
| 35 and older | 414.4 (43.2) | 15.3 (25.4) | 3.7 (41.8) |  | 327.5 (50.8) | 88.4 (52.6) | 27.1 (48.7) |  |
| **Marital Status** |  |  |  | 0.008 |  |  |  | 0.286 |
| Divorced, Separated, Widowed, Missing | 121.0 (12.6) | 6.9 (11.5) | 0.5 (5.9) |  | 22.0 (3.4) | 4.5 (2.7) | 0.3 (0.5) |  |
| Currently married | 766.0 (79.8) | 40.1 (66.6) | 3.6 (41.1) |  | 552.6 (85.7) | 136.7 (81.3) | 44.8 (80.4) |  |
| Currently single | 73.1 (7.6) | 13.2 (21.9) | 4.6 (53.0) |  | 70.5 (10.9) | 27.0 (16.1) | 10.7 (19.1) |  |
| **Education level** |  |  |  | 0.507 |  |  |  | 0.624 |
| No Schooling | 129.8 (13.7) | 3.0 (5.1) | 0.0 (0.0) |  | 49.4 (7.8) | 8.0 (4.8) | 3.6 (6.5) |  |
| Primary/Secondary | 774.6 (81.7) | 56.3 (94.9) | 8.8 (100.0) |  | 549.2 (87.3) | 151.4 (91.6) | 47.1 (85.3) |  |
| Post-secondary | 44.1 (4.7) | 0.0 (0.0) | 0.0 (0.0) |  | 30.8 (4.9) | 5.9 (3.6) | 4.5 (8.1) |  |
| **Household wealth**: Poorest quantile | 111.3 (11.6) | 11.4 (19.0) | 0.0 (0.0) | 0.436 | 106.7 (16.5) | 22.3 (13.2) | 4.5 (8.1) | 0.32 |
| **Occupation** |  |  |  | 0.033 |  |  |  | 0.001 |
| Informal sector: low risk | 738.2 (76.9) | 41.8 (69.5) | 4.1 (46.6) |  | 475.4 (73.7) | 93.9 (55.8) | 28.0 (50.2) |  |
| Informal sector: high risk | 56.5 (5.9) | 1.9 (3.1) | 0.0 (0.0) |  | 32.9 (5.1) | 5.6 (3.3) | 3.5 (6.3) |  |
| Formal sector | 102.6 (10.7) | 10.0 (16.7) | 0.1 (1.3) |  | 99.3 (15.4) | 55.5 (33.0) | 19.6 (35.1) |  |
| **HIV infection** | 138.9 (14.5) | 17.4 (29.0) | 1.6 (18.8) | 0.001 | 65.1 (10.1) | 24.8 (14.8) | 12.3 (22.0) | 0.001 |
| **Migration in 2015-2016: Any** | 73.9 (7.7) | 18.2 (30.3) | 3.1 (35.2) | 0.005 | 70.9 (11.0) | 32.3 (19.2) | 14.6 (26.2) | 0.004 |
| Any internal migration | 73.9 (7.7) | 18.2 (30.3) | 3.1 (35.2) | 0.005 | 69.6 (10.8) | 32.2 (19.1) | 14.6 (26.2) | 0.003 |
| Intra-District/Sub-county | 54.2 (5.6) | 10.1 (16.8) | 0.0 (0.0) | 0.078 | 17.3 (2.7) | 18.5 (11.0) | 6.9 (12.3) | 0.002 |
| Inter-District/Sub-county | 20.3 (2.1) | 8.1 (13.5) | 3.1 (35.2) | <0.001 | 53.4 (8.3) | 14.8 (8.8) | 8.2 (14.7) | 0.308 |
| Any international migration | NA | NA | NA |  | 2.3 (0.4) | 0.3 (0.2) | 0.0 (0.0) | 0.648 |

Notes: Data are weighted; column percentages shown; missing data excluded for education level and occupation.

**Table A.2: Characteristics Associated with Number of Sexual Partnerships in Past 6 Months, by Sex**

|  | **Women** | | | | **Men** | | | |
| --- | --- | --- | --- | --- | --- | --- | --- | --- |
| **Characteristic** | **Number of sexual partners in past 6 months** | | | | **Number of sexual partners in past 6 months** | | | |
|  | **0 or 1** | **2** | **3 or more** | ***p*** | **0 or 1** | **2** | **3 or more** | ***p*** |
| Weighted n | 960.1 | 60.1 | 8.8 |  | 645.2 | 168.2 | 55.7 |  |
| **Region** |  |  |  | 0.150 |  |  |  | 0.008 |
| Kenya - Western | 516.6 (53.8) | 43.8 (72.9) | 5.2 (58.9) |  | 316.4 (49.0) | 121.2 (72.1) | 41.8 (75.2) |  |
| Uganda - Eastern | 196.5 (20.5) | 0.3 ( 0.5) | 0.0 ( 0.0) |  | 143.6 (22.2) | 17.3 (10.3) | 6.4 (11.5) |  |
| Uganda - South Western | 247.0 (25.7) | 16.0 (26.7) | 3.6 (41.1) |  | 185.3 (28.7) | 29.7 (17.6) | 7.4 (13.4) |  |
| **Age Band** |  |  |  | 0.063 |  |  |  | 0.524 |
| 16-24 | 202.7 (21.1) | 22.3 (37.1) | 4.6 (53.0) |  | 121.6 (18.8) | 39.9 (23.7) | 14.0 (25.2) |  |
| 25-34 | 343.0 (35.7) | 22.6 (37.5) | 0.5 ( 5.2) |  | 196.2 (30.4) | 39.9 (23.7) | 14.5 (26.1) |  |
| 35 and older | 414.4 (43.2) | 15.3 (25.4) | 3.7 (41.8) |  | 327.5 (50.8) | 88.4 (52.6) | 27.1 (48.7) |  |
| **Marital Status** |  |  |  | 0.008 |  |  |  | 0.286 |
| Divorced, Separated, Widowed, Missing | 121.0 (12.6) | 6.9 (11.5) | 0.5 ( 5.9) |  | 22.0 ( 3.4) | 4.5 ( 2.7) | 0.3 ( 0.5) |  |
| Currently married | 766.0 (79.8) | 40.1 (66.6) | 3.6 (41.1) |  | 552.6 (85.7) | 136.7 (81.3) | 44.8 (80.4) |  |
| Currently single | 73.1 ( 7.6) | 13.2 (21.9) | 4.6 (53.0) |  | 70.5 (10.9) | 27.0 (16.1) | 10.7 (19.1) |  |
| **Education level** |  |  |  | 0.507 |  |  |  | 0.624 |
| No Schooling | 129.8 (13.7) | 3.0 ( 5.1) | 0.0 ( 0.0) |  | 49.4 ( 7.8) | 8.0 ( 4.8) | 3.6 ( 6.5) |  |
| Primary/Secondary | 774.6 (81.7) | 56.3 (94.9) | 8.8 (100.0) |  | 549.2 (87.3) | 151.4 (91.6) | 47.1 (85.3) |  |
| Post-secondary | 44.1 ( 4.7) | 0.0 ( 0.0) | 0.0 ( 0.0) |  | 30.8 ( 4.9) | 5.9 ( 3.6) | 4.5 ( 8.1) |  |
| **Household wealth**: Poorest quantile | 111.3 (11.6) | 11.4 (19.0) | 0.0 ( 0.0) | 0.436 | 106.7 (16.5) | 22.3 (13.2) | 4.5 ( 8.1) | 0.32 |
| **Occupation** |  |  |  | 0.033 |  |  |  | 0.001 |
| Informal sector: low risk | 738.2 (76.9) | 41.8 (69.5) | 4.1 (46.6) |  | 475.4 (73.7) | 93.9 (55.8) | 28.0 (50.2) |  |
| Informal sector: high risk | 56.5 ( 5.9) | 1.9 ( 3.1) | 0.0 ( 0.0) |  | 32.9 ( 5.1) | 5.6 ( 3.3) | 3.5 ( 6.3) |  |
| Formal sector | 102.6 (10.7) | 10.0 (16.7) | 0.1 ( 1.3) |  | 99.3 (15.4) | 55.5 (33.0) | 19.6 (35.1) |  |
| **HIV infection** | 138.9 (14.5) | 17.4 (29.0) | 1.6 (18.8) | 0.001 | 65.1 (10.1) | 24.8 (14.8) | 12.3 (22.0) | 0.001 |
| **Mobility in Past 6 Months** |  |  |  |  |  |  |  |  |
| Any labor-related travel, past 6 mo. | 24.8 ( 2.6) | 4.4 ( 7.3) | 0.3 ( 3.6) | 0.257 | 107.7 (16.7) | 38.9 (23.1) | 13.9 (24.9) | 0.168 |
| No. nights away, labor-related travel | 0.81 (8.76) | 5.00 (20.33) | 0.07 (0.39) | 0.070 | 4.31 (17.74) | 7.69 (29.26) | 11.17 (30.13) | 0.104 |
| Any non-labor-related travel, past 6 mo. | 532.7 (55.5) | 47.4 (78.9) | 7.6 (87.1) | 0.011 | 180.2 (27.9) | 75.3 (44.8) | 27.8 (49.9) | 0.002 |
| No. nights away, non-work travel | 5.09 (12.63) | 12.77 (21.20) | 24.95 (27.40) | 0.035 | 2.23 (8.21) | 4.26 (12.33) | 1.82 (3.03) | 0.188 |
| **Sexual behavior, Past 6 Months** |  |  |  |  |  |  |  |  |
| Any concurrent sex partners, past 6 months | 0.0 ( 0.0) | 44.2 (73.8) | 8.3 (94.6) | <0.001 | 0.0 ( 0.0) | 129.8 (77.2) | 51.7 (97.6) | <0.001 |
| Any high risk sex partners, past 6 mo. | 54.8 ( 5.7) | 27.7 (46.1) | 2.6 (29.8) | <0.001 | 31.4 ( 4.9) | 37.1 (22.0) | 30.4 (54.7) | <0.001 |

Notes: Data are weighted; column percentages shown; missing data excluded for education level and occupation.

**Table A.3: Associations of Measures of Mobility with Concurrent Sexual Partnerships Over a Two-Year Period (2015-2016) [Full model output]**

|  | **Adjusted Odds Ratio, any Concurrent Sexual Partnership, 2015-2016, ALL** | | | | | | | | **Adjusted Odds Ratio, any Concurrent Sexual Partnership, 2015-2016, MEN** | | | | | | | | **Adjusted Odds Ratio, any Concurrent Sexual Partnership, 2015-2016, WOMEN** | | | | | | | | | | |  |
| --- | --- | --- | --- | --- | --- | --- | --- | --- | --- | --- | --- | --- | --- | --- | --- | --- | --- | --- | --- | --- | --- | --- | --- | --- | --- | --- | --- | --- |
| **Characteristic** | **Model 1: All** | | | | **Model 2: All** | | | | **Model 1: Men** | | | | **Model 2: Men** | | | | **Model 1: Women** | | | | **Model 2: Women** | | | | | | |  |
|  | **aOR** | ***p*** | **a95% CI** | | **aOR** | ***p*** | **a95% CI** | | **aOR** | ***p*** | **a95% CI** | | **aOR** | ***p*** | **a95% CI** | | **aOR** | ***p*** | **a95% CI** | | **aOR** | | ***p*** | | **a95% CI** | | |  |
| Gender: female (ref.: male) | 0.23 | 0.000 | 0.17 | 0.31 | 0.23 | 0.000 | 0.17 | 0.31 | NA |  |  |  | **NA** |  |  |  | **NA** |  |  |  | | **NA** | |  | |  |  | |
| Region (%) |  |  |  |  |  |  |  |  |  |  |  |  |  |  |  |  |  |  |  |  | |  | |  | |  |  | |
| Kenya - Western (ref.) |  |  |  |  |  |  |  |  |  |  |  |  |  |  |  |  |  |  |  |  | |  | |  | |  |  | |
| Uganda - Eastern | 0.35 | 0.000 | 0.21 | 0.56 | 0.33 | 0.000 | 0.21 | 0.54 | 0.39 | 0.001 | 0.22 | 0.68 | 0.38 | 0.001 | 0.22 | 0.67 | 0.14 | 0.007 | 0.03 | 0.58 | | 0.12 | | 0.004 | | 0.03 | 0.52 | |
| Uganda - South Western | 0.67 | 0.039 | 0.46 | 0.98 | 0.64 | 0.022 | 0.44 | 0.94 | 0.61 | 0.038 | 0.38 | 0.97 | 0.64 | 0.060 | 0.40 | 1.02 | 0.88 | 0.663 | 0.50 | 1.56 | | 0.75 | | 0.333 | | 0.41 | 1.35 | |
| Age Band |  |  |  |  |  |  |  |  |  |  |  |  |  |  |  |  |  |  |  |  | |  | |  | |  |  | |
| 16-24 (ref.) |  |  |  |  |  |  |  |  |  |  |  |  |  |  |  |  |  |  |  |  | |  | |  | |  |  | |
| 25-34 | 0.59 | 0.019 | 0.38 | 0.92 | 0.57 | 0.014 | 0.37 | 0.90 | 0.70 | 0.247 | 0.39 | 1.28 | 0.69 | 0.213 | 0.38 | 1.24 | 0.49 | 0.044 | 0.25 | 0.98 | | 0.50 | | 0.056 | | 0.25 | 1.02 | |
| 35 or older | 0.80 | 0.300 | 0.52 | 1.23 | 0.76 | 0.203 | 0.49 | 1.16 | 1.10 | 0.747 | 0.61 | 1.97 | 1.03 | 0.908 | 0.58 | 1.84 | 0.46 | 0.025 | 0.23 | 0.91 | | 0.45 | | 0.022 | | 0.22 | 0.89 | |
| Marital Status |  |  |  |  |  |  |  |  |  |  |  |  |  |  |  |  |  |  |  |  | |  | |  | |  |  | |
| Married/divorce/separated/widowed (ref.) |  |  |  |  |  |  |  |  |  |  |  |  |  |  |  |  |  |  |  |  | |  | |  | |  |  | |
| Currently single | 1.29 | 0.314 | 0.79 | 2.12 | 1.26 | 0.360 | 0.77 | 2.07 | 1.18 | 0.621 | 0.61 | 2.28 | 1.17 | 0.640 | 0.61 | 2.25 | 1.83 | 0.111 | 0.87 | 3.86 | | 1.79 | | 0.131 | | 0.84 | 3.80 | |
| Occupation |  |  |  |  |  |  |  |  |  |  |  |  |  |  |  |  |  |  |  |  | |  | |  | |  |  | |
| Informal sector: low risk (ref.) |  |  |  |  |  |  |  |  |  |  |  |  |  |  |  |  |  |  |  |  | |  | |  | |  |  | |
| Informal sector: high risk | 1.12 | 0.667 | 0.66 | 1.89 | 1.13 | 0.647 | 0.67 | 1.91 | 1.42 | 0.243 | 0.79 | 2.56 | 1.46 | 0.211 | 0.81 | 2.63 | 0.42 | 0.25 | 0.10 | 1.84 | | 0.43 | | 0.266 | | 0.10 | 1.90 | |
| Formal sector | 1.36 | 0.067 | 0.98 | 1.88 | 1.37 | 0.057 | 0.99 | 1.91 | 1.45 | 0.053 | 1.00 | 2.11 | 1.45 | 0.052 | 1.00 | 2.11 | 1.18 | 0.648 | 0.58 | 2.37 | | 1.24 | | 0.55 | | 0.62 | 2.47 | |
| missing | 1.04 | 0.903 | 0.59 | 1.80 | 1.06 | 0.842 | 0.61 | 1.84 | 0.71 | 0.389 | 0.33 | 1.54 | 0.70 | 0.365 | 0.33 | 1.51 | 1.25 | 0.582 | 0.56 | 2.82 | | 1.20 | | 0.663 | | 0.53 | 2.75 | |
|  |  |  |  |  |  |  |  |  |  |  |  |  |  |  |  |  |  |  |  |  | |  | |  | |  |  | |
| HIV infection | 1.50 | 0.003 | 1.15 | 1.95 | 1.52 | 0.002 | 1.16 | 1.98 | 1.22 | 0.213 | 0.89 | 1.66 | 1.24 | 0.176 | 0.91 | 1.69 | 2.32 | 0.002 | 1.36 | 3.97 | | 2.36 | | 0.002 | | 1.38 | 4.05 | |
| Migrations in 2015-2016 |  |  |  |  |  |  |  |  |  |  |  |  |  |  |  |  |  |  |  |  | |  | |  | |  |  | |
| Any internal migration (ref.: none) | 1.60 | 0.005 | 1.15 | 2.23 |  |  |  |  | 1.50 | 0.042 | 1.01 | 2.21 |  |  |  |  | 1.99 | 0.027 | 1.08 | 3.68 | |  | |  | |  |  | |
| Inter-District/Sub-county (ref.: none) |  |  |  |  | 1.63 | 0.420 | 0.67 | 2.65 |  |  |  |  | 1.43 | 0.228 | 0.80 | 2.56 |  |  |  |  | | 2.93 | | 0.021 | | 1.18 | 7.28 | |

Notes: Multilevel mixed-effects logistic regression models adjusted for clustering at community level. Intradistrict migration in past two years not shown; not found to be independently associated with concurrency in pooled adjusted model.

**Table A.4: Associations of Measures of Mobility with Higher Risk Sexual Partnerships Over a Two-Year Period (2015-2016) [Full model output]**

|  | **Adjusted Odds Ratio, any Higher Risk Sexual Partnership, 2015-2016** | | | | | | | | | | | |
| --- | --- | --- | --- | --- | --- | --- | --- | --- | --- | --- | --- | --- |
| **Characteristic** | **ALL** | | | | **MEN** | | | | **WOMEN** | | | |
|  | **aOR** | ***p*** | **a95% CI** | | **aOR** | ***p*** | **a95% CI** | | **aOR** | ***p*** | **a95% CI** | |
| Gender: female (ref.: male) | 0.77 | 0.071 | 0.58 | 1.02 | NA |  |  |  | NA |  |  |  |
| Region (%) |  |  |  |  |  |  |  |  |  |  |  |  |
| Kenya - Western (ref.) |  |  |  |  |  |  |  |  |  |  |  |  |
| Uganda - Eastern | 0.05 | 0.000 | 0.01 | 0.17 | 0.07 | 0.000 | 0.01 | 0.29 | 0.03 | 0.001 | 0.00 | 0.25 |
| Uganda - South Western | 0.97 | 0.902 | 0.60 | 1.56 | 1.31 | 0.376 | 0.72 | 2.37 | 0.68 | 0.126 | 0.42 | 1.11 |
| Age Band |  |  |  |  |  |  |  |  |  |  |  |  |
| 16-24 (ref.) |  |  |  |  |  |  |  |  |  |  |  |  |
| 25-34 | 0.82 | 0.440 | 0.51 | 1.35 | 0.36 | 0.004 | 0.18 | 0.73 | 1.82 | 0.121 | 0.85 | 3.90 |
| 35 or older | 0.92 | 0.726 | 0.57 | 1.49 | 0.37 | 0.005 | 0.19 | 0.75 | 2.50 | 0.014 | 1.20 | 5.20 |
| Marital Status |  |  |  |  |  |  |  |  |  |  |  |  |
| Married/divorce/separated/widowed (ref.) |  |  |  |  |  |  |  |  |  |  |  |  |
| Currently single | 4.48 | 0.000 | 2.74 | 7.32 | 3.72 | 0.000 | 1.85 | 7.49 | 3.98 | 0.000 | 1.85 | 8.56 |
| Occupation |  |  |  |  |  |  |  |  |  |  |  |  |
| Informal sector: low risk (ref.) |  |  |  |  |  |  |  |  |  |  |  |  |
| Informal sector: high risk | 0.77 | 0.407 | 0.42 | 1.43 | 1.10 | 0.807 | 0.50 | 2.44 | 0.60 | 0.350 | 0.20 | 1.76 |
| Formal sector | 1.42 | 0.059 | 0.99 | 2.05 | 1.65 | 0.035 | 1.04 | 2.62 | 0.85 | 0.595 | 0.46 | 1.56 |
| missing | 0.86 | 0.615 | 0.49 | 1.53 | 1.07 | 0.877 | 0.46 | 2.46 | 0.93 | 0.870 | 0.41 | 2.14 |
| **Household wealth**: Poorest quantile | 1.78 | 0.001 | 1.25 | 2.52 | 1.92 | 0.006 | 1.20 | 3.05 | 1.76 | 0.045 | 1.01 | 3.06 |
| HIV infection | 2.02 | 0.000 | 1.49 | 2.74 | 1.74 | 0.013 | 1.13 | 2.68 | 2.84 | 0.000 | 1.80 | 4.50 |
| Migrations in 2015-2016 |  |  |  |  |  |  |  |  |  |  |  |  |
| Any internal migration (ref.: none) | 1.32 | 0.119 | 0.93 | 1.88 | 1.53 | 0.061 | 0.98 | 2.40 | 1.07 | 0.835 | 0.56 | 2.03 |

Notes: Multilevel mixed-effects logistic regression models adjusted for clustering at community level. Inter- and Intra-district migration in past two years not shown; not found to be independently associated with concurrency in pooled adjusted model.
