# Supplementary figures and images for: Population mobility associated with higher risk sexual behaviour in eastern African communities participating in a Universal Testing and Treatment trial
Source: J Int AIDS Soc. 2018 Jul 19;21(Suppl Suppl 4):e25115. doi: 10.1002/jia2.25115 (PMC6053476; doi:10.1002/jia2.25115)

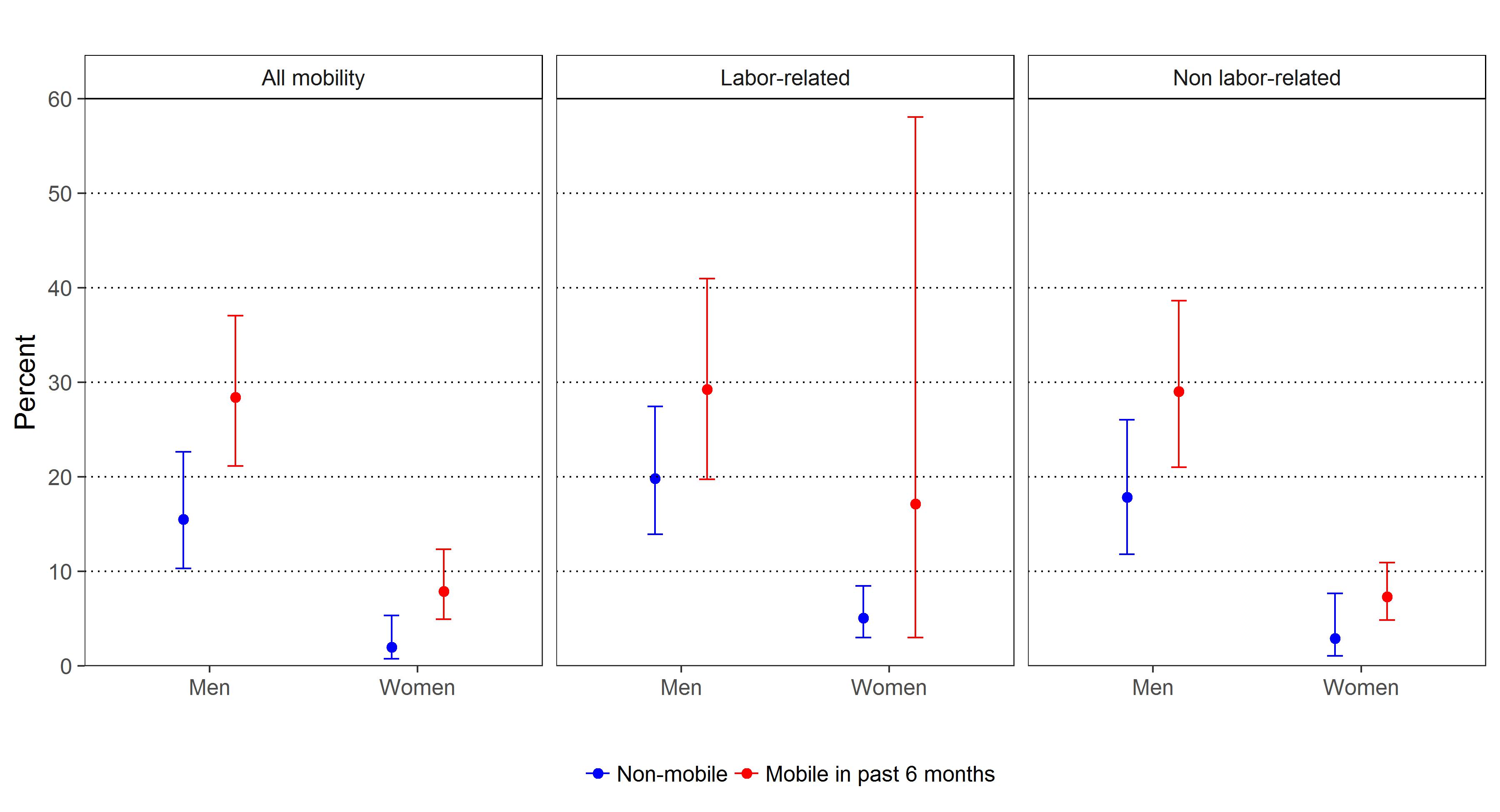

Supplement: Supplementary file 2 — Figure S1.Prevalence of Sexual Partnership Concurrency in Past 6 Months, by Sex and Mobility in Past 6 Months, by Type of Mobility. [file JIA2-21-e25115-s002.jpg]
